# Membrane Vesicle-Mediated Delivery of Antibacterial Lipopeptides by *Pseudoalteromonas piscicida*

**DOI:** 10.1021/acschembio.5c01016

**Published:** 2026-02-27

**Authors:** Ololade S. Gbadebo, Arvie Grace Masibag, Margaret E. Rosario, Ruolin He, Yan-Song Ye, Marta Gomez-Chiarri, Qihao Wu, David C. Rowley

**Affiliations:** † Department of Biomedical & Pharmaceutical Sciences, College of Pharmacy, 4260 The University of Rhode Island, Kingston, Rhode Island 02881, United States; ‡ Biological Sciences Department, College of Arts and Sciences, Cavite State University, Indang, Cavite 4122, Philippines; § Bioinformatics Group, 593528 Wageningen University, Droevendaalsesteeg 1, Wageningen 6708 PB, Netherlands; ∥ Department of Pharmaceutical Sciences, 6614 University of Pittsburgh, Pittsburgh, Pennsylvania 15261, United States; ⊥ Department of Fisheries, Animal and Veterinary Sciences, The University of Rhode Island, Kingston, Rhode Island 02881, United States

## Abstract

Bacterial membrane vesicles (MVs) are natural delivery systems for biomolecules, such as enzymes and nucleic acids, but their role in transporting specialized metabolites is less understood. Many microbial metabolites are lipophilic and poorly water-soluble, raising questions about how they perform ecological functions in aquatic environments. Here, we demonstrate that *Pseudoalteromonas piscicida* JC3, a marine bacterium with probiotic potential, packages lipophilic depsipeptides known as bromoalterochromides (BACs) into outer membrane vesicles. Untargeted metabolomics and molecular networking identified six known and two previously unknown BACs, while targeted LC–MS/MS localized BACs to MVs and cells, with no detection in culture supernatants. Structure elucidation of a new analogue, bromoalterochromide E/E′, was achieved through isolation and spectroscopic analysis, including modified Marfey’s analysis to determine amino acid composition and chirality. Functional assays showed that BAC-loaded MVs exhibit antibacterial activity against *Staphylococcus aureus* and the marine pathogen *Vibrio anguillarum*, linking vesicle-mediated metabolite delivery to microbial competition. These findings highlight MVs as transporters of lipophilic natural products and suggest their potential as natural drug delivery vehicles in clinical and aquaculture settings.

## Graphical abstract

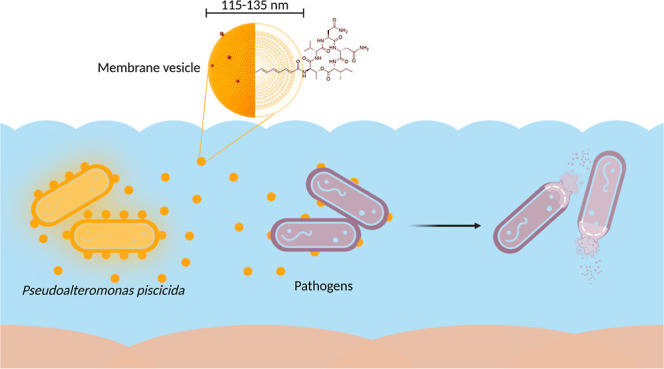

## Introduction

All cells have the potential to produce extracellular vesicles (EVs), making them a fundamental product of life.^1^ EVs from eukaryotes are generally called exosomes, while those from prokaryotes are commonly termed membrane vesicles (MVs). Bacterial MVs are usually within the size range of 20–400 nm. Gram-negative bacteria produce outer membrane vesicles (OMVs) to package and deliver sensitive macromolecules, including nucleic acids and enzymes, that mediate cell–cell interactions, including communication, competition, and horizontal gene transfer.^2–6^ Less is known about the role that the OMVs play in the exchange of specialized metabolites. However, it can be expected that OMVs would provide an advantageous “drug delivery” system for transporting lipophilic compounds and delivering them at sufficient concentrations to elicit a dose-related response in the receiving cell.^7^ Furthermore, OMVs can protect encapsulated cargo from environmental damage, such as hydrolysis,^7,8^ act as decoys for antimicrobial peptides and bacteriophages,^9,10^ and carry enzymes such as β-lactamases that contribute to antibiotic resistance.^11^ In addition, OMVs have been linked with antimicrobial effects against bacterial competitors^7^ and may stimulate the production of otherwise “silent” metabolites in recipient cells.^12^ Bacterial membrane vesicles are formed through blebbing and endolysin-triggered explosive cell lysis (Figure 1), which influences their composition.^5^ Despite contributing only a fraction of the small colloidal particles in seawater, their ability to package compounds in high local concentration suggests that they serve specific functions in marine microbiomes.^13^

**Figure 1:**
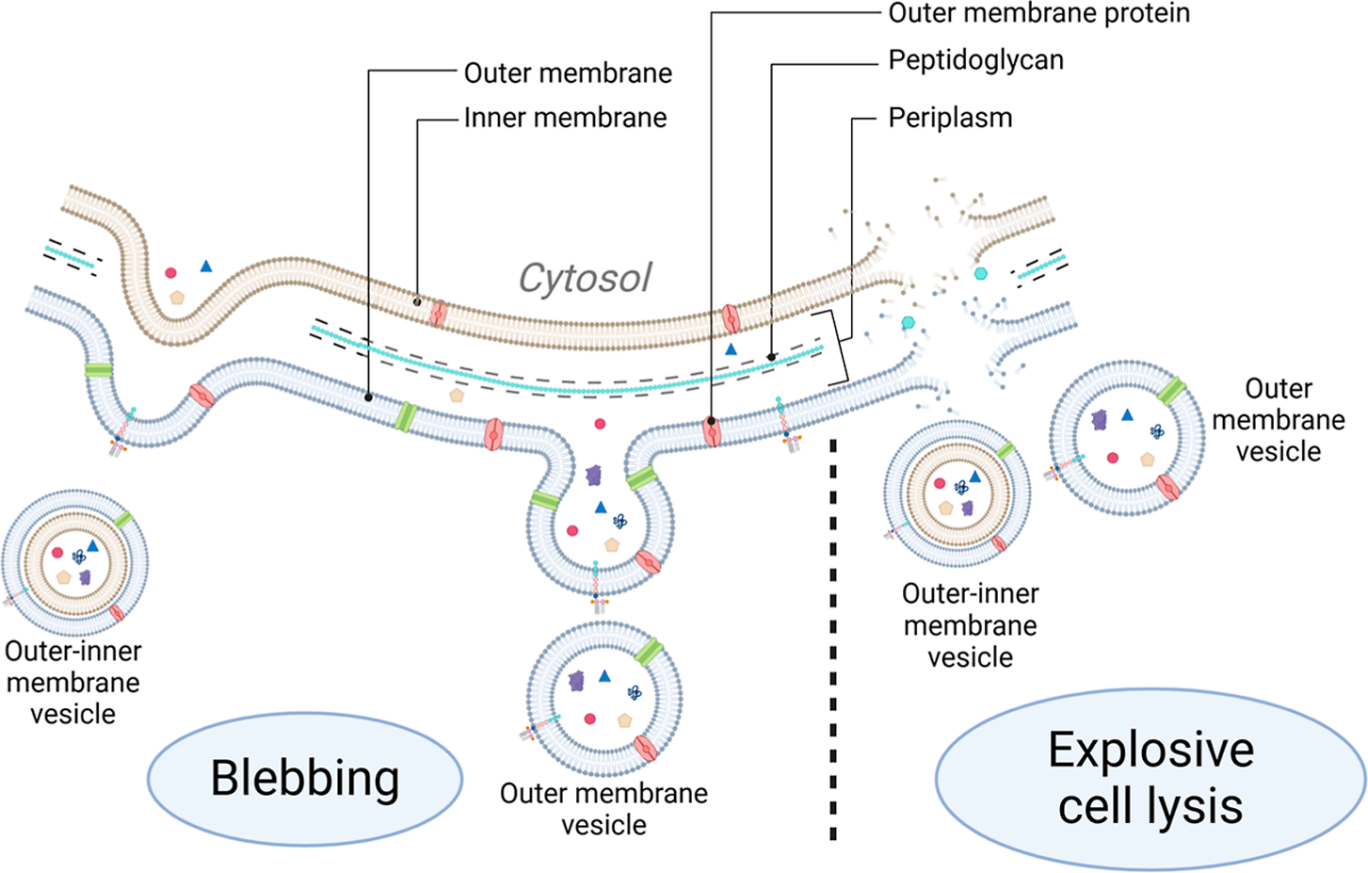
Biogenic pathways of Gram-negative bacterial membrane vesicles. Gram-negative bacterial MVs are formed either through blebbing or phage-induced explosive lysis. Irregular peptidoglycan formation or the buildup of hydrophobic molecules in the periplasm can accumulate beneath the outer membrane, forming a bleb that eventually detaches to form outer membrane vesicles (OMVs). Weakening of the peptidoglycan layer and bulging of the inner membrane through the periplasm can result in the formation of outer–inner membrane vesicles (OIMVs). Vesicles may also form via autophage-induced explosive lysis of the bacterial cell, with the resulting fragments reassembling to produce explosive outer membrane vesicles (EOMVs) and explosive outer–inner membrane vesicles.

Tripartite interactions among pathogens, probiotic bacteria, and hosts often involve the exchange of specialized metabolites. Marine probiotics may produce antimicrobial metabolites to suppress pathogen growth and promote host health.^14,15^ This potential is particularly evident in the Gram-negative genus *Pseudoalteromonas,* which is known for producing diverse bioactive compounds, including alkaloids, polyketides, nonribosomal peptides (NRPs), and bacteriocins.^16^ To date, more than 60 *Pseudoalteromonas* species have been reported,^17^ with most exhibiting pigmentation due to the production of conjugated specialized metabolites.^18^ Notable examples of antimicrobial metabolites originating from this genus include koromicins and tetrabromopyrrole from *Pseudoalteromonas peptidolytica*;^19^ violacein, pentabromopseudilin, indolmycin, and thiomarinols from *Pseudoalteromonas luteoviolacea*;^20^ a tambjamine from *P. tunicata*;^21^ and prodigiosins from *Pseudoalteromonas rubra*.^22^ Although the bioactivity of these metabolites is well documented, their mechanisms of delivery, particularly via membrane vesicles, remain unexplored.

In this study, we investigated specialized metabolites packaged within the OMVs produced by the putative probiotic, *Pseudoalteromonas piscicida* JC3. *P. piscicida* strain JC3 was isolated from whiteleg shrimp (*Litopenaeus vannamei*), and genomic sequencing indicated its capacity for diverse microbial interactions.^23^ Genomic analysis further revealed multiple biosynthetic gene clusters (BGCs) for specialized metabolites, including a nonribosomal peptide synthetase linked with the biosynthesis of bromoalterochromides (BACs).^24,25^ BACs are yellow-pigmented lipopeptides composed of a cyclic depsipeptide core and a conjugated brominated aromatic side chain. Variants differ in lipid side chain length and amino acid substitutions within the peptide sequence.^26,27^ BACs exhibit diverse bioactivities, including antibacterial, antifungal, antiprotozoal, and nitric oxide inhibitory activities.^27^

We hypothesized that *P. piscicida* JC3 produced BACs and that these lipophilic metabolites are secreted via vesicle packaging rather than direct diffusion into the aqueous environment.^27–29^ Here, we show that *P. piscicida* JC3 packages BACs as MV cargo, with concentrations depending on cultivation conditions. Untargeted metabolomic analysis revealed six known and two previously unidentified BACs were contained within isolated OMVs. Furthermore, higher BAC concentrations correlated with increased antimicrobial activity against bacterial pathogens, supporting a role for *P. piscicida* MVs in microbial competition by delivering toxic cargoes. This study demonstrates that the metabolomic analysis of bacterial vesicles can provide insights into lipophilic metabolite trafficking between cells and highlights the potential to discover new bioactive natural products.

## Results

### Isolation and Characterization of membrane Vesicles from *P. piscicida* JC3

To enable their metabolomic characterization and the assessment of their biological roles, we established a workflow for MV isolation from broth cultures of *P. piscicida* JC3 (Figure 2A). Cultures were grown under shaken or static conditions for 24 h (exponential growth phase) or 48 h (stationary growth phase), and membrane vesicles were collected by ultracentrifugation (Figure 2A). MVs were characterized by nanoparticle tracking analysis (NTA) and transmission electron microscopy (TEM). TEM imaging (Figure 2B) revealed that the vesicles from all growth conditions were predominantly bounded by single membranes, consistent with outer membrane vesicles (OMVs).^30^

**Figure 2:**
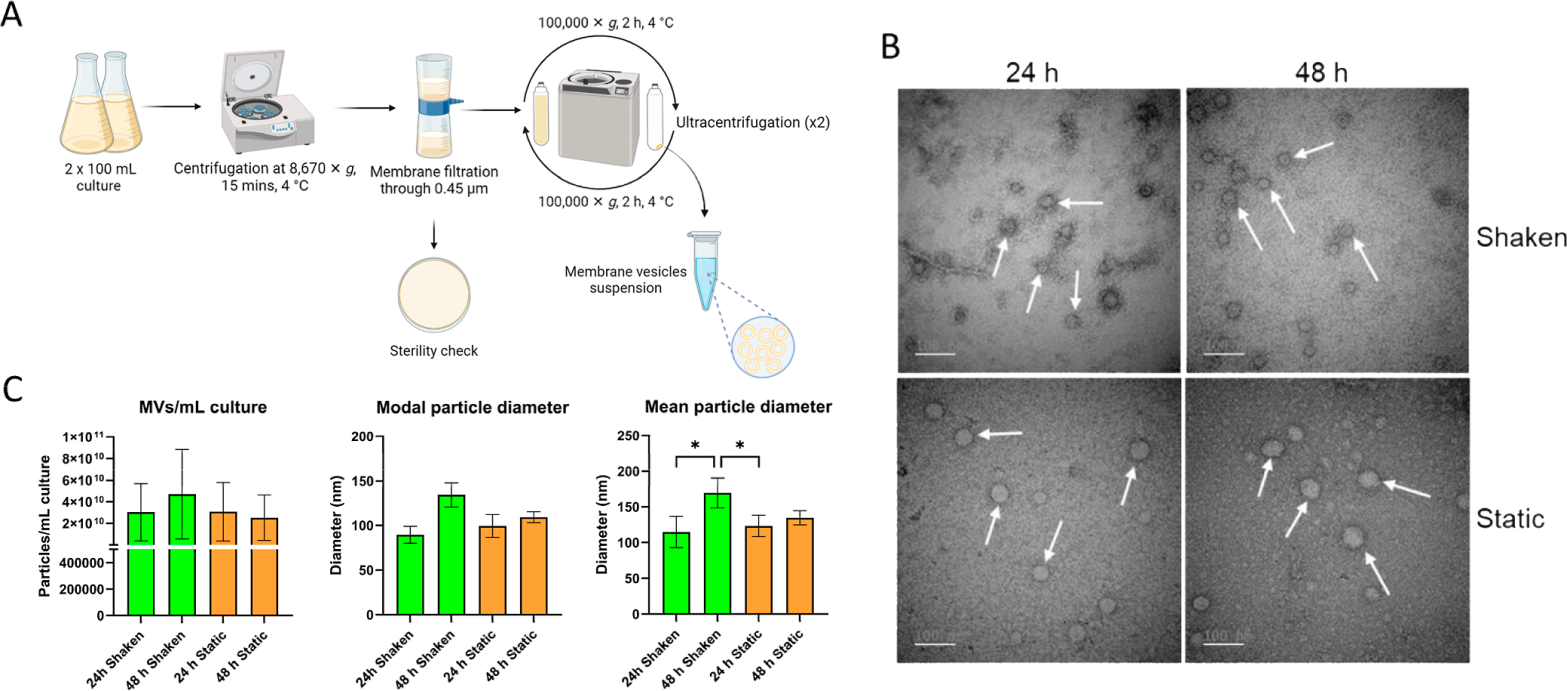
(A) Experimental workflow for the isolation of membrane vesicles from bacterial culture. (B) Electron micrographs confirming outer membrane vesicles isolated from *P. piscicida* JC3 cultures (*D* = 100 nm scale). (C) MVs from the shaken and static cultures grown for 24 and 48 h were analyzed for particle size distribution and concentration using Nanoparticle Tracking Analysis. Data are presented as mean ± SEM of biological triplicates. Data were analyzed using one-way analysis of variance (ANOVA), followed by Tukey’s post hoc pairwise comparisons (*p < 0.05).

NTA quantified vesicle abundance under the various cultivation conditions. The highest concentration (4.69 × 10^10^ ± 4.13 × 10^10^ particles/mL) was observed in 48 h shaken cultures. Other concentrations were 3.04 × 10^9^ ± 2.65 × 10^9^ (24 h shaken), 3.08 × 10^10^ ± 2.72 × 10^10^ (24 h static), and 2.52 × 10^10^ ± 2.1 × 10^10^ particles/mL (48 h static) (Figure 2C). Vesicle size distributions also varied: the modal diameters were 89 ± 9 (24 h shaken), 134 ± 14 (48 h shaken), 99 ± 13 (24 h static), and 109 ± 6 nm (48 h static). Mean diameters followed the same trend (Figure 2C). MV concentrations showed no statistically significant differences between the exponential and stationary growth phases (ANOVA, *p* > 0.05) (Figure S1). However, vesicles harvested at 48 h tended to be larger, suggesting either a time-dependent increase in size or aggregation, which may be influenced by factors such as temperature, pH, vesicle composition, and ionic strength.^31^

### Metabolomic Profiling of Cell and MV Extracts Reveals BAC Cargo

To investigate specialized metabolite content, cells and MVs from shaken or static cultures were separated using sequential centrifugation and ultracentrifugation and then freeze-dried and extracted with methanol. Static cultures yielded less biomass than shaken ones, but MV and cell pellets from static conditions showed greater pigmentation (Figure S2 and Table S1).

Genome mining with antiSMASH^32^ identified 23 biosynthetic gene clusters (BGCs), including those predicted to encode nonribosomal peptides (NRPs), polyketides (PKs), and ribosomally synthesized and post-translationally modified peptides (RiPP) (Table S2). Initial metabolomic profiling of the extracts using HPLC-UV revealed greater complexity and abundance in the samples from static cultures (Figure 3A,B). Untargeted LC–MS/MS metabolomics profiling supported this trend, with UpSet plot analysis revealing that cell extracts contained more unique features than those from MVs (Figure 3C). Cell and MV extracts from static cultures displayed larger metabolomes (set size) than those from their shaken counterparts (Figure 3C). In total, 39 features were shared across all conditions.

**Figure 3:**
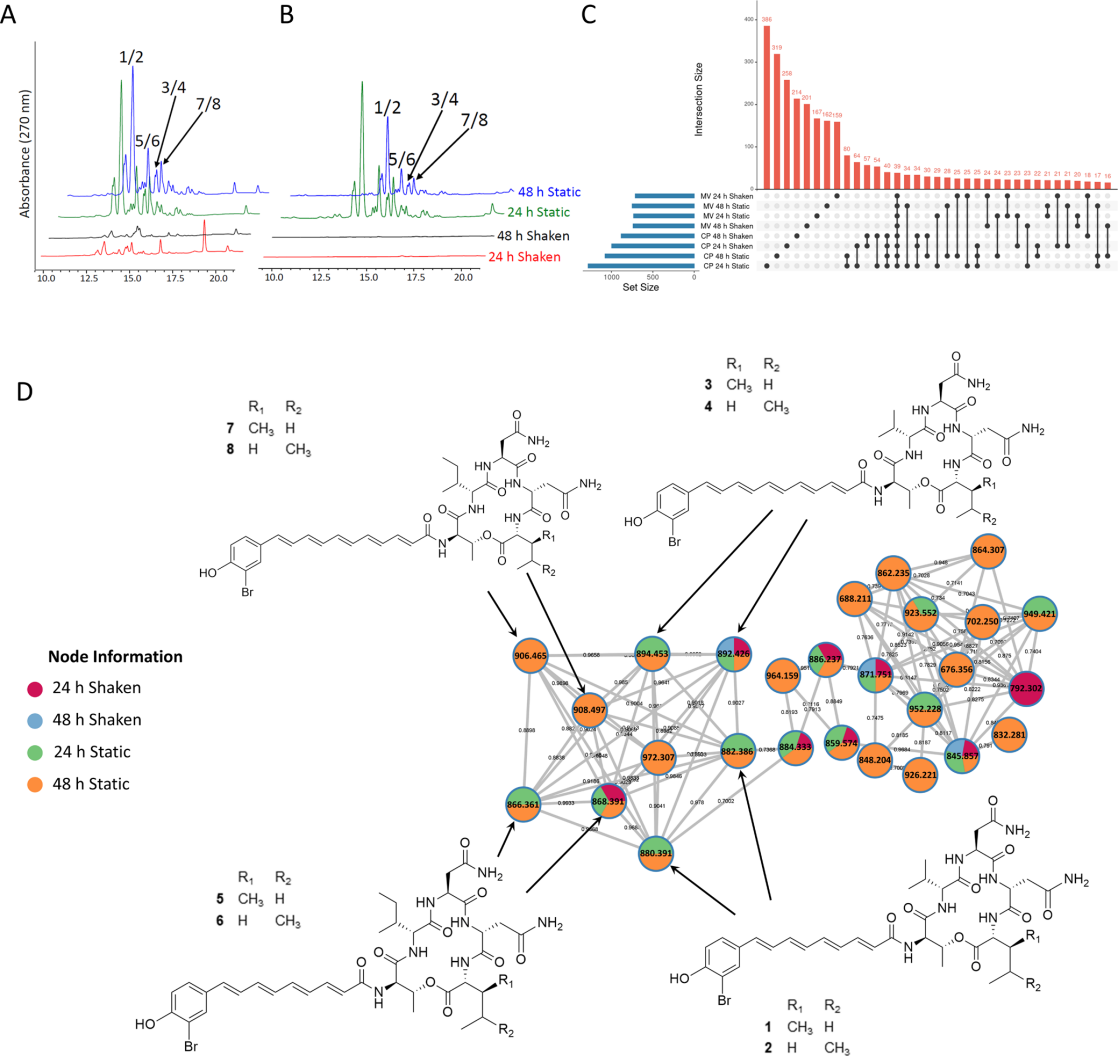
Metabolomic profiling of MV and cell pellet extracts. Normalized HPLC-UV chromatograms (λ = 270 nm) of (A) MV extracts and (B) cell extracts from static cultures contain more metabolites than those from shaken cultures. Samples were prepared at 10 mg mL^–1^ with an injection volume of 10 μL. (C) The UpSet plot shows the number of unique metabolite features detected in the untargeted LC–MS/MS data. The intersection size denotes the number of unique features detected in each extract, whereas the set size denotes the total number of metabolites detected. (D) Molecular networks reveal the presence of bromoalterochromides in the MV extracts. Arrows connect the compounds represented by the nodes to their respective chemical structures. Pie charts in the nodes show the relative abundance of compounds at each *m*/*z* across different MV extracts. The networks were created in the GNPS platform and exported to Cytoscape software (version 3.9.1) for processing.

MS/MS feature-based molecular networking using the Global Natural Products Social Molecular Networking (GNPS) web platform^33^ identified both known and previously uncharacterized bromoalterochromides (BACs) (Figures 3D, S3, and S4), consistent with the presence of the BAC BGC identified in the genome. Bromine isotopic patterns in the LC/MS data further supported this finding (Figure S5). BACs were most abundant in static culture extracts, consistent with their greater visible pigmentation and HPLC-UV profiles (Figures 3A,B, S2, and S6). MS^2^ fragmentation patterns supported the annotation of the cyclic peptides bromoalterochromides A/A′ (**1**/**2**), B/B′ (**3**/**4**), and bromoalterochromides D/D′ (**5**/**6**),^25,26,28,29^ and revealed two new analogs, which we designate bromoalterochromides E/E′ (**7**/**8**) (Figures 3, S4, and S10).

### BACs Are Localized within *P. piscicida* Membrane Vesicles and Cells but Are Undetected in Culture Supernatants

To delineate the patterns of BAC concentrations within cells, MVs, and supernatants, we quantified the BACs in their methanolic extracts using a triple quadrupole mass spectrometer coupled with ultra-HPLC. Isolated BAC A/A′ was utilized to generate a standard curve because commercial standards are unavailable (Figure S11). Peak areas were used to compare the minor BAC constituents. The LC–MS/MS parameters were optimized using the BAC A/A′ standard. All transitions were tested individually and collectively using the BAC A/A′ standard, revealing that the response signals were additive (Figure S12). Therefore, one adduct (*m*/*z* = 846.2) was selected as the quantifier ion, while the others served as qualifiers. Analysis of extracts from shaken cultures showed that BACs were more localized in the MVs than in the cells, whereas BACs were more abundant in cells than in the MVs from static cultures. A one-way ANOVA test showed statistical significance in the BAC contents of MV and cell extracts grown under shaking and static conditions (Figure 4). Notably, BACs were not detected in the supernatant extracts from any of the cultures (Figure 4). Regardless of the source, BAC concentrations followed this relative order in abundance: BAC A/A′ > B/B′ > D/D′ > E/E′ (Figures 4 and S13). Additionally, their retention times followed this order: BAC A/A′ < D/D′ < B/B′ < E/E′, reflecting the longer lipophilic chains in BAC B/B′ and BAC E/E′ (Figure S13).

**Figure 4:**
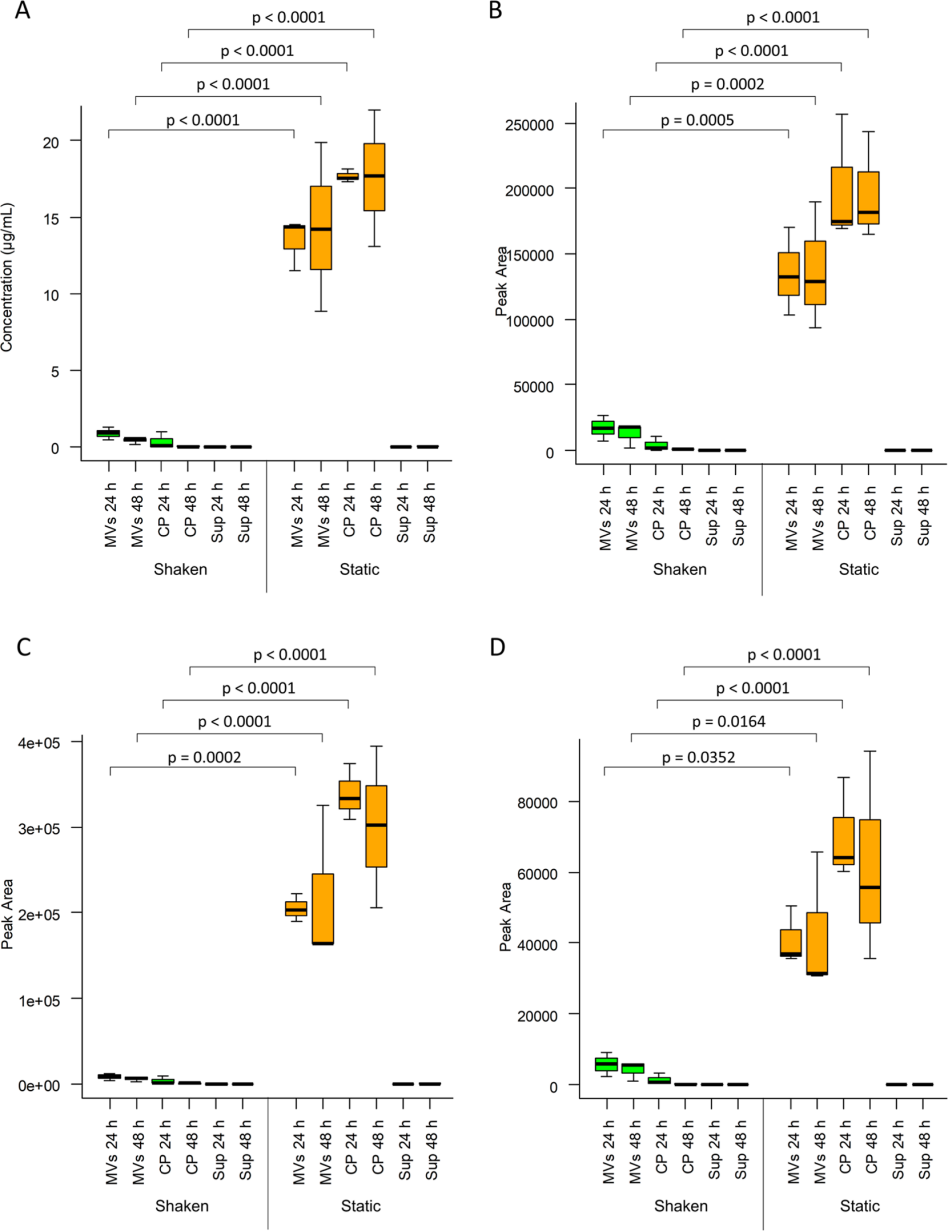
Bromoalterochromides are localized in MVs and cells of *P. piscicida* JC3. Plots show the targeted quantitation of (A) BAC A/A′, (B) BAC B/B′, (C) BAC D/D′, and (D) BAC E/E′ in MV, cell pellet, and supernatant extracts derived from the shaken and static cultures. BAC A/A′ concentrations in μg/mL were determined using a standard curve. The concentrations of bromoalterochromides B/B′, D/D′, and E/E′ are indicated by peak areas. BACs were not detected in MV-free culture supernatants. The boxes display the interquartile range, while the black lines inside the boxes indicate the median values from three biological replicates. The whiskers indicate the minimum and maximum values. Statistics were analyzed using one-way analysis of variance (ANOVA) followed by Tukey’s correction. MVs = membrane vesicle extracts, CP = cell pellet extracts, Sup = cell- and MV-free supernatant extracts.

### Membrane Vesicles Deliver Antimicrobial Cargo

Using a spot plate assay, we first evaluated the antibacterial activity of MVs from *P. piscicida* JC3 against the fish pathogen *Vibrio anguillarum* NB10. MV suspensions were adjusted to 3.72 × 10^9^ MVs/mL in phosphate-buffered saline (PBS) before adding 10 μL aliquots directly onto an agar surface inoculated with NB10. Disks loaded with 10 μg of BAC A/A′ or ciprofloxacin served as positive controls, whereas PBS served as the negative control. MVs from shaken cultures showed no detectable activity (Figure 5A). In contrast, MVs from static cultures created clear zones of inhibition, indicating antibacterial activity. BAC A/A′ demonstrated only weak inhibition in comparison to that of ciprofloxacin (Figure 5A).

**Figure 5:**
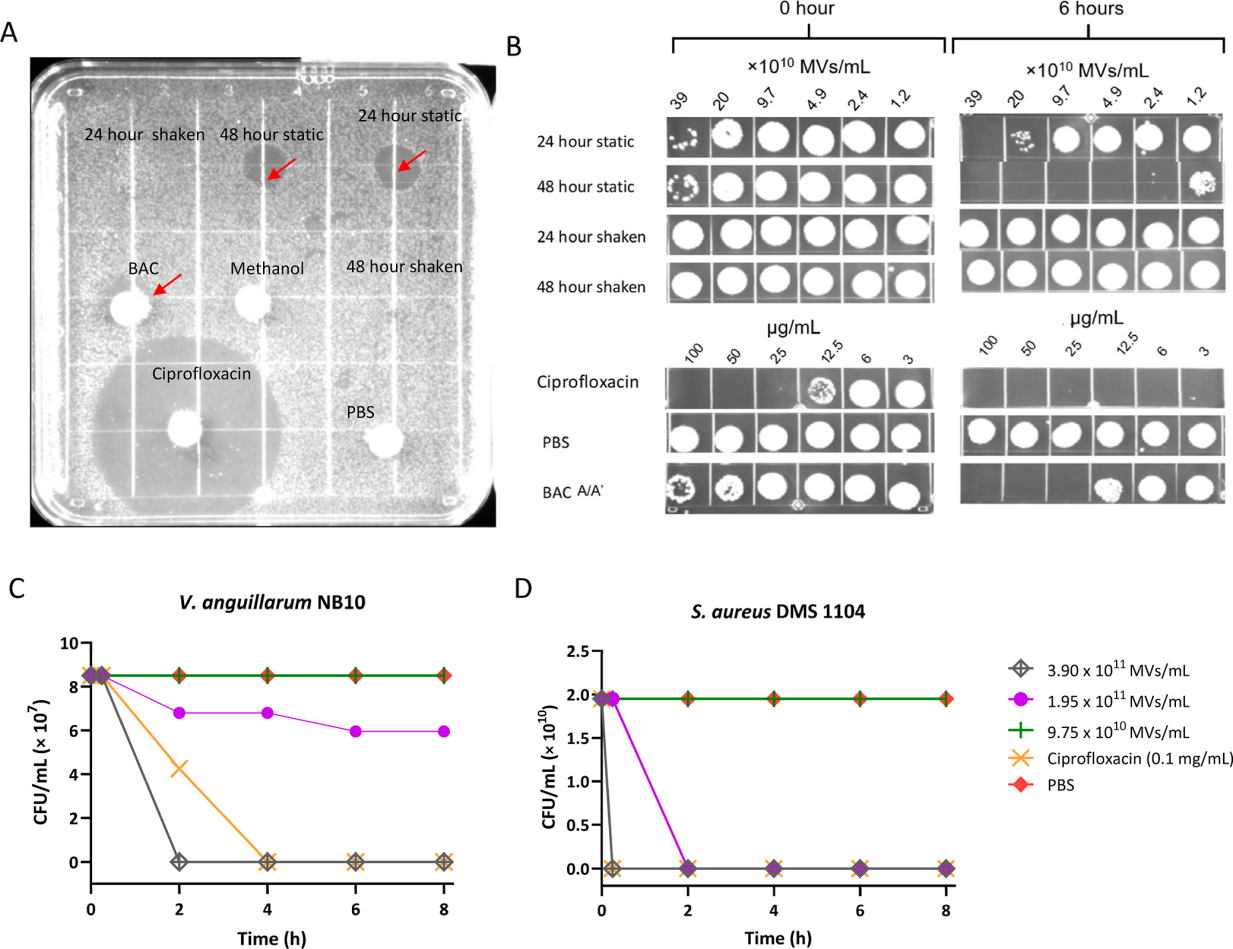
Antimicrobial activity of *P. piscicida* JC3MVs against *V. anguillarum* NB10 and *S. aureus* DMS 1104. (A) Image shows an agar plate inoculated with *V. anguillarum* NB10 and incubated at 27 °C for 24 h. Test samples included 10 μL of MV suspensions (3.72 × 10^9^ MVs/mL in PBS) or sterile disks loaded with 10 μg of BAC A/A′ or ciprofloxacin. (B) Antibacterial time-killing activity of MVs against *S. aureus* DMS 1104. Bacterial cultures were treated with serially diluted MVs, BAC A/A′, and ciprofloxacin (control). Culture aliquots (5 μL) were spotted onto agar at 0 and 6 h time points and incubated for 24 h. (C) Colony-forming units of *V. anguillarum* NB10 and (D) *S. aureus* DMS 1104 cultures over 8 h following treatment with MVs collected from 48 h static cultures.

Using a microbroth dilution assay, we measured the antibacterial activities of serial dilutions of MVs against *V. anguillarum* NB10, the shrimp pathogen *Vibrio parahaemolyticus* PSU5579, the oyster pathogen *Vibrio coralliilyticus* RE22, and the opportunistic human pathogen *Staphylococcus aureus* DSM1104 (Figure 5B). MVs derived from shaken cultures showed no detectable antimicrobial activity. In contrast, MVs from static cultures displayed rapid bactericidal effects within 2 h. *S. aureus* DSM1104 was inhibited across nearly all concentrations tested (2.4 × 10^10^ to 3.9 × 10^11^ MVs/mL), while *V. anguillarum* NB10 was inhibited at higher concentrations (≥2.0 × 10^11^ MVs/mL) (Figures 5B and S17). Overall, MVs collected from 48 h static cultures exhibited the strongest activity. Purified BAC A/A′ also inhibited both test organisms. Neither MVs nor BAC A/A′ showed activity against *V. parahaemolyticus* PSU5579 or *V. coralliilyticus* RE22 (Figure S17).

To examine the dose-dependent antibacterial effects, we tested the viability of *V. anguillarum* NB10 and *S. aureus* DSM1104 over time in response to different concentrations of 48 h static MVs. The highest concentration (3.9 × 10^11^ MVs/mL) resulted in a faster onset of inhibition against *V. anguillarum* NB10 than ciprofloxacin (2 h), while both treatments achieved similar inhibition onset (∼25 min) against *S. aureus* DSM1104 (Figure 5C,D).

Due to the potent antibacterial effects observed for the 48 h static MVs, we next determined their minimum inhibitory concentration (MIC) and minimum bactericidal concentration (MBC) against *V. anguillarum* NB10, *S. aureus* DSM1104, *S. aureus* ATCC 35556, and three clinical isolates of methicillin-resistant *S. aureus* (MRSA). Ciprofloxacin and BAC A/A′ served as positive controls. All *S. aureus* strains were more sensitive to JC3 MVs than *V. anguillarum* NB10 (Table 1). Among these, *S. aureus* ATCC 35556 showed the highest susceptibility (MIC = 2.4 × 10^10^ MVs/mL), while the MRSA isolates were one or two dilutions less sensitive (Table 1).

**Table 1:** Minimum Inhibitory (MIC) and Minimum Bactericidal (MBC) Concentrations of JC3 Membrane Vesicles against *Vibrio anguillarum* and *Staphylococcus aureus*

	VA NB10^a^		SA DSM 1104^a^		SA ATCC 35556		MRSA L17^b^		MRSA L32^b^		MRSA L44^b^	
	MIC	MBC	MIC	MBC	MIC	MBC	MIC	MBC	MIC	MBC	MIC	MBC
JC3 MVs (E10 MVs/mL)	20	20	4.9	9.8	2.4	4.9	9.8	9.8	4.9	9.8	4.9	9.8
BAC A/A′ (μM)	4.6	4.6	4.6	4.6	18	18	37	37	18	37	37	37
Ciprofloxacin (μM)	1.2	2.4	2.4	2.4	2.4	4.8	>300	>300	>300	>300	>300	>300

a VA = *Vibrio anguillarum*, SA = *Staphylococcus aureus*, MRSA = methicillin-resistant *S. aureus.*

b Clinical isolates.

### Isolation and Structure Elucidation of Bromoalterochromide E/E′ (**7**/**8**)

Analysis of LC–MS/MS data from MVs and cell pellet extracts revealed the presence of two potential new BAC analogs, designated compounds **7**/**8** (Figures 3 and S3). Interestingly, previous metabolomic studies of *P. piscicida* strains T1lg65 and JCM 20779 hinted at similar, previously undescribed BACs, although isolation and structural confirmation were not performed.^26,34^ To further establish the identities of these compounds, we undertook large-scale cultivation of *P. piscicida* JC3 for subsequent purification and spectroscopic studies.

Lyophilized cell pellets from 14 L of *P. piscicida* JC3 cultured statically for 8 days were extracted with methanol and dichloromethane, and the resulting crude was fractionated by sequential reversed-phase C18 chromatography to afford 0.6 mg of compounds **7**/**8**, the least abundant of the BACs characterized by this study. High-resolution MS analysis of compounds **7**/**8** showed [M + H]^+^ *m*/*z* of 884.3130, agreeing with the calculated *m*/*z* of 884.3188 for C_41_H_55_BrN_7_O_10_^+^ (Figure S18). Comparison of MS^2^ spectra from all isolated BACs showed that compound **7**/**8** produced unique fragment ions with *m*/*z* of 525.1360, 639.1782, and 753.2211 (Figures 6A and S19), highlighting its structural differences. BAC A/A′ and D/D′ share an identical side chain but differ by substitution of Leu/Ile for Val in the cyclic peptide, accounting for a *m*/*z* difference of 14 amu. We noted that the *m*/*z* of BAC E/E′ similarly differed from that of BAC B/B′ by 14 amu, consistent with these two peptides also sharing the same side chain but varying in their amino acid sequences. Inspection of the MS^2^ spectra of BAC B/B′ and BAC E/E′ confirmed a swap of Val and Leu/Ile in their respective amino acid sequences (Figures S19 and S20).

**Figure 6:**
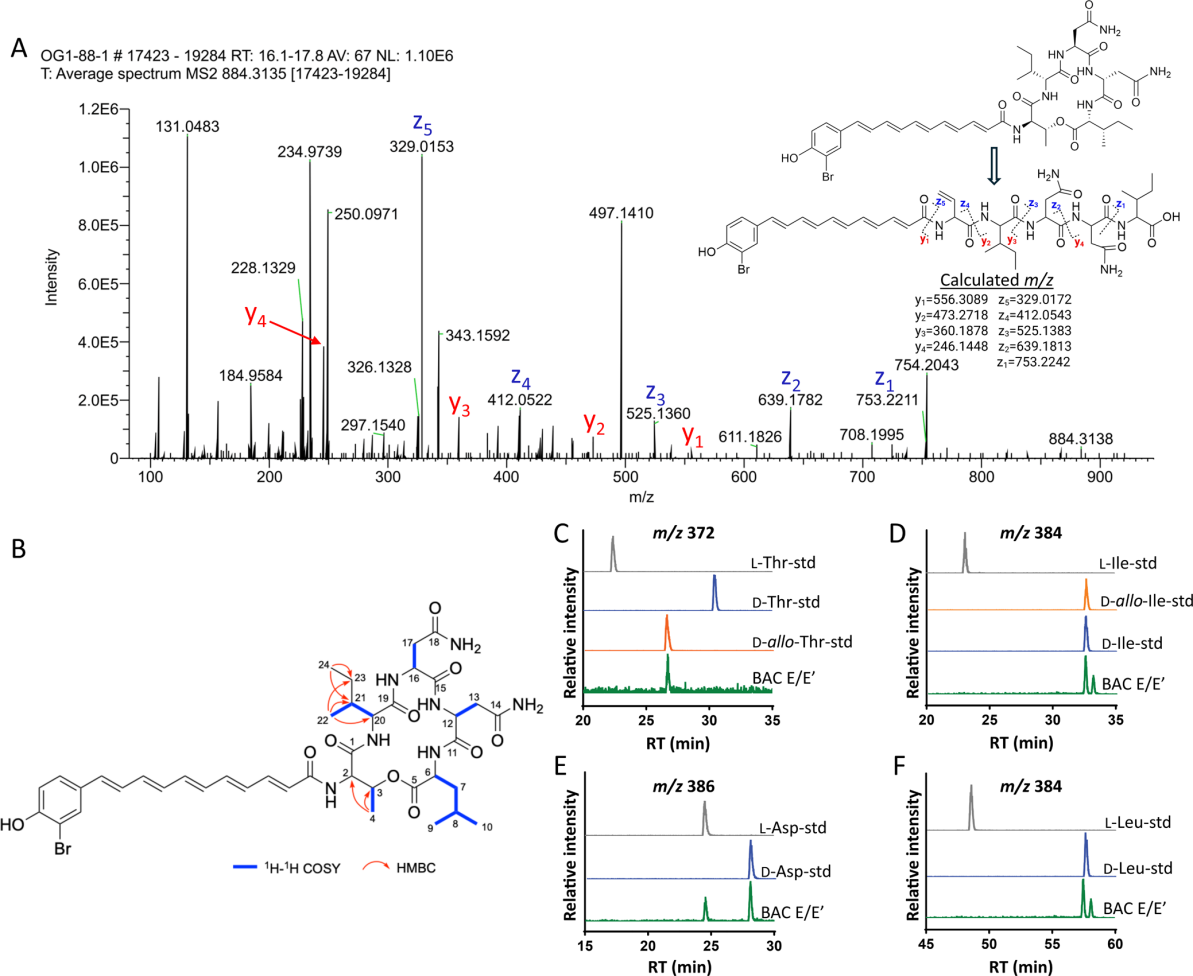
Structure elucidation of BAC E/E′. (A) MS^2^ fragmentation of protonated BAC E/E′ shows opening of the cyclic peptide chain followed by characteristic cleavages between peptide bonds. (B) 2D NMR correlations of BAC E/E′. (C–F) Marfey’s analysis comparing L-FDLA derivatives of hydrolyzed BAC E/E′ amino acids to amino acid standards of threonine (C), isoleucine (D), aspartic acid (E), and leucine (F). Asparagine is converted to aspartic acid during peptide hydrolysis.

Analysis of the 1D and 2D NMR spectra revealed the presence of five amino acid residues: Thr, Ile, two Asn, and Leu (Figure 6B). The threonine residue was identified based on the ^1^H–^1^H COSY correlations between H-4 and H-3, supported by HMBC correlations from Me-4 to C-3 and C-2. The leucine residue was assigned based on a ^1^H–^1^H COSY spin system comprising Me-9, Me-10, H-8, H_2_-7, and H-6. Two asparagine residues were recognized via the ^1^H–^1^H COSY correlations of H-12/H_2_-13 and H-16/H_2_-17. Finally, the isoleucine residue was confirmed by key ^1^H–^1^H COSY correlations between Me-22, H-21, and H-20, along with HMBC correlations from Me-24 to C-23 and C-21 and from Me-22 to C-21, C-20, and C-23 (Figure S25).

The stereochemistry of the BAC amino acids was determined using a modified C_18_ Marfey’s analysis.^35^ Asn was converted to Asp under the hydrolytic conditions, prompting the use of Asp standards for the analysis. LC/MS analysis of BAC E/E′ hydrolysate derivatized with 1-fluoro-2,4-dinitrophenyl-5-l-leucinamide (L-FDLA) revealed the presence of d-*allo*-Thr, both l-Asn and d-Asn, and D-configurations for Leu and Ile residues (Figure 6C–F). These results established the amino acid sequence for the cyclic peptide of **7**/**8** to be d-*allo*-Thr–d-Leu/d-Ile/d-*allo*-Ile–l-Asn/d-Asn–l-Asn/d-Asn–d-Leu/d-Ile/d-*allo*-Ile. Using the previously reported JC3 draft genome,^23^ we conducted a bioinformatic analysis of the putative BAC biosynthetic gene cluster (BGC). This analysis revealed epimerization domains within modules predicted to incorporate Thr and Leu–Ile residues, consistent with the observed stereochemistry. However, gaps in the draft sequence prevented the characterization of the two Asn modules (Figure S26). Based on prior bioinformatic studies of a bromoalterochromide BGC from *P. piscicida*,^27^ we tentatively assign l-Asn and d-Asn to Asn-1 and Asn-2, respectively.

## Discussion

This study provides the first evidence that *Pseudoalteromonas* membrane vesicles (MVs) serve as vehicles for delivering lipophilic specialized metabolites. Previous chemical investigations of *Pseudoalteromonas* MVs have primarily focused on macromolecular cargo, including proteins, lipids, and DNA, with roles in environmental adaptation and gene transfer.^6,36^ The data here expand upon these findings by demonstrating that *P. piscicida* JC3 packages lipodepsipeptides known as the bromoalterochromides (BACs) into MVs, suggesting a mechanism for their delivery between cells in marine microbial ecosystems.

Owing to the lipophilicity of the BACs, free diffusion in aqueous environments is unlikely to be an effective mechanism for achieving competitive interactions with nearby cells. We hypothesized that *P. piscicida* JC3 solves this problem by packaging BACs within MVs. Toyofuku and co-workers previously proposed that MVs serve as quantal delivery systems for transporting bioactive cargo at concentrations sufficient to achieve a minimum effective dose. Within marine ecosystems, hydrophobic molecules within MVs may travel longer distances from the producing cells while maintaining concentrations high enough to elicit an effect. In this regard, bacterial MVs act much like liposomes for the delivery of poorly soluble drugs.^7^

We found that BACs were localized within MVs and cell pellets with no detectable presence in MV-free supernatants, supporting MV packaging rather than passive release. Notably, MVs from static cultures exhibited higher BAC concentrations and more potent antimicrobial activity than those from shaken cultures, showing that growth conditions influence the MV composition. BAC abundance was also higher in cell extracts from static cultures, suggesting a potential role for BACs in biofilms or as a stress response to oxygen depletion. Interestingly, BACs were more localized in MVs than in cells from shaken cultures. Further studies under varying environmental conditions may yield insights into the stimuli that drive BAC production.

Bromoalterochromides are widely produced within the *Pseudoalteromonas* genus^19,26,28,29,37,38^ and have been reported for their antibacterial, antifungal, and antiprotozoal activities and inhibition of nitric oxide production activities. Untargeted metabolomic analysis of the MV and cell pellet extracts revealed a suite of BACs produced by JC3, including a previously uncharacterized analog, BAC E/E′, which was subsequently isolated from large-scale cultures and structurally characterized using MS, NMR, and modified C_18_ Marfey’s analyses. BAC E/E′ differs from BAC B/B′ by substitution of valine with leucine/isoleucine in the cyclic peptide sequence. We note the prior MS detection of a metabolite with an *m*/*z* consistent with BAC E/E′,^26,34^ indicating that biosynthesis of BAC E/E′ is not likely limited to JC3. The discovery of BAC E/E′ expands the structural diversity of BACs and highlights the use of MV metabolomics for natural product discovery.

The antimicrobial activity against *S. aureus*, an opportunistic human pathogen, encourages further investigation of MVs as antibiotic adjuvants and drug delivery tools. Huang et al. previously found that levofloxacin packaged within MVs produced by *Acinetobacter baumannii* was more effective at killing enterotoxin-producing *Escherichia coli* in a mouse model than the free drug.^39^ OMVs may fuse with the outer membranes of Gram-negative bacterial cells, thus delivering their molecular cargo and bypassing the stringent selection of porins for compound transport.^40,41^ In this context, the use of OMVs serves as a natural drug delivery system to enhance the penetration of antibacterial agents into Gram-negative cells. Further mechanistic understanding of the role of OMVs in microbial interactions may inform novel approaches to improve the clinical delivery and efficacy of antibacterial drugs.

*Pseudoalteromonas* spp. have been studied as promising probiotic additives to prevent infectious disease outbreaks in aquaculture systems.^42–44^ The delivery of antimicrobial metabolites by MVs is an unexplored mechanism for competitive interactions between probiotic and pathogenic bacteria. *P. piscicida* JC3 MVs demonstrated activity against the *V. anguillarum* NB10 strain, a fish pathogen, indicating a potential role for limiting its growth in an aquaculture system. The lack of BAC A/A′ and JC3 MVs activity against *Vibrio parahaemolyticus* PSU5579 and *V. coralliilyticus* RE22 may reflect resistance mechanisms or differential selectivity for MV delivery, warranting further investigation.

Potential synergistic effects between specialized metabolites and enzymes copackaged within MVs warrant further investigation. MVs from *Citrobacter*, *Enterobacter*, *Escherichia*, *Klebsiella*, *Morganella*, *Proteus*, *Pseudomonas*, *Salmonella*, and *Shigella* isolates have been shown to lyse various Gram-positive and Gram-negative bacteria.^7^ MVs tethered to the outer surface of *P. piscicida* can transfer lytic enzymes to other cells upon contact.^42^ Concomitant delivery of antimicrobial specialized metabolites and enzymes, such as proteases and hydrolases, may accelerate cell killing in recipient cells.^3,7,45^ Future proteomic analysis of JC3 MVs may provide insights into the potential combinatorial effects of BACs and enzymes to achieve competitive outcomes with other bacteria.

## Experimental Section

### Bacterial Strains and Growth Conditions

Marine bacterial cultures (*P. piscicida* JC3, *V. parahaemolyticus* PSU5579, *V. coralliilyticus* RE22, *V. anguillarum* NB10) were cultured in YP30 broth (0.1% yeast, 0.5% peptone from meat, 3% Instant Ocean) and incubated at 27 °C and 175 rpm. *S. aureus* DSM 1104, *S. aureus* ATCC 35556, and methicillin-resistant *S. aureus* L17, *S. aureus* L32, and *S. aureus* L44 were cultivated in Mueller–Hinton medium (Millipore, Sigma-Aldrich) at 37 °C. Strains L17, L32, and L44 are clinical isolates obtained from patients at the Providence Veterans Affairs Medical Center and were kindly provided by Dr. Kerry LaPlante.^46,47^ All bacterial strains were maintained in media with 25% glycerol at −80 °C.

### Growth Curve Determination

A 1 mL portion of an overnight culture of *P. piscicida* JC3 was introduced into 100 mL of YP30 media. Cultures were grown at 27 °C with shaking at 175 rpm. Absorbance measurements (λ = 600 nm) were made on aliquots collected at 30 min intervals for 6 h, at 3 h intervals until 12 h, at 12 h intervals until 48 h, and at 76 h. Cultures were grown in triplicate.

### Membrane Vesicle Isolation and Purification

A 10 mL overnight culture of *P. piscicida* JC3 was diluted 1:100 into sterile YP30 broth and incubated at 27 °C for 24 or 48 h under static or shaking (175 rpm) conditions. Membrane vesicles (MVs) from *P. piscicida* JC3 were isolated as previously described with modifications.^48^ Bacterial cultures were centrifuged (Beckman Coulter Avanti-J-E Centrifuge, JA-10 rotor) at 8670*g* and 4 °C for 15 min, and the supernatants were filtered through a 0.45 μm filter. The cell pellets were collected and stored at −20 °C. The filtrate (10 μL) was spread onto a YP30 agar plate to confirm sterility. The cell-free supernatants were ultracentrifuged (Beckman Coulter Optima L-100 XP Centrifuge, 70.1 Ti rotor) at 33,000 rpm and 4 °C for 2 h. The MV-free supernatant was transferred and stored at 4 °C. The MV pellet was resuspended in phosphate-buffered saline (PBS), divided into two equal volumes, and ultracentrifuged using the same conditions. After the PBS was discarded, the resulting MV pellets were resuspended in 300 μL of Milli-Q water (for metabolomic analysis) or 1× PBS with 0.1% DMSO and stored at −20 °C.

### Nanoparticle Tracking Analysis

MV concentrations and particle size distributions were quantified using a Nanosight NS500 nanoparticle tracking analyzer (Malvern Instruments), calibrated with nanosphere standard beads (Thermo Scientific, USA). Particle per frame values (20–100 particles/frame) were pretested to ensure ranges recommended by the manufacturer. Videos captured were used to determine the mean, mode, median, and estimated concentrations for each particle size. Triplicate samples for each growth condition were analyzed.

### Transmission Electron Microscopy

Briefly, 5 μL of a 100-fold diluted MV suspension was placed on a 300-mesh carbon-coated grid (Ted Pella, USA) and cross-linked with 2.5% glutaraldehyde for 2 min, washed with sterile water for 2.5 min, and negatively stained with 2% uranyl acetate (Electron Microscopy Sciences, USA) for 2 min. Micrographs were obtained with TEM using a JEM-2100 80 KeV instrument at the Rhode Island Consortium for Nanoscience and Nanotechnology, Kingston, RI.

### Sample Extraction

Frozen MV suspensions were lyophilized overnight in 2 mL centrifuge tubes (Labconco FreeZone freeze-dryer). The resulting MV solids were then extracted once with 600 μL of methanol, vortexed for 1 min, sonicated for 1 min, and centrifuged (Eppendorf centrifuge 5418) at 7000 rpm for 10 min to pellet insoluble constituents. The CH_3_OH extract was transferred, concentrated in vacuo (Savant SpeedVac SPD1030, ThermoScientific), and stored at −20 °C until further use. Bacterial cell pellets were lyophilized, extracted with 10 mL of CH_3_OH using sonication, and centrifuged at 7000 rpm for 10 min to pellet insoluble cell debris. The CH_3_OH fractions were similarly concentrated in vacuo and stored at −20 °C. MV-free culture supernatants were extracted using 30 mg/3 mL Strata-X 33 μm polymeric solid phase extraction columns (Phenomenex). The columns were sequentially preconditioned with 6 mL of CH_3_OH and 6 mL of H_2_O before 8 mL of MV-free supernatants. The columns were then washed with 6 mL of 10% aqueous CH_3_OH to remove polar media constituents. Finally, the columns were eluted with 1 mL of CH_3_OH, which was collected, concentrated in vacuo, and stored at −20 °C.

### Metabolomics Analysis of MV Extracts

UV-HPLC analyses were conducted using a Shimadzu Prominence-I LC-2030C 3D HPLC equipped with a DAD unit and a Kinetex 2.6 μm C18 column fitted with a SecurityGuard ULTRA Cartridge (Phenomenex, USA). The column was maintained at 40 °C. The mobile phases consisted of 0.1% formic acid in H_2_O (A) and 0.1% formic acid in CH_3_CN (B). Samples were dissolved in CH_3_OH at 1 mg mL^–1^ and analyzed with an injection volume of 10 μL and a flow rate of 0.6 mL/min. Chromatography method: the mobile phase was maintained at 5% B for 5 min, linearly increased to 100% B over 12 min, held at 100% B for 13 min, and then returned to 5% B over 1 min. Untargeted metabolomic analyses were performed using an LTQ XL mass spectrometer (Thermo Scientific, USA) in combination with a Dionex Ultimate 3000 HPLC system (Thermo Scientific, USA) equipped with an autosampler and a DAD. The sample concentration, injection volume, column, and mobile phases were the same as those above, except that the column and flow rate were maintained at 30 °C and 0.4 mL/min, respectively. Method: the mobile phase was maintained at 5% B for 5 min, linearly increased to 100% B over 15 min, held at 100% B for 10 min, returned to 5% B over 1 min, and held at 5% B for 9 min. Data were acquired in positive ionization mode at a capillary temperature of 323 °C and a spray voltage of 3.5 kV.

Targeted metabolomic analyses were conducted using an Agilent 1290 Infinity LC system coupled to an Agilent 6740B triple quadrupole (QQQ) mass spectrometer with an Agilent Jet Stream Source electrospray ionizer. Instrument conditions, transitions, and MRM parameters are provided in Table S3. For quantification of BAC A/A′, a standard curve was generated within the concentration range 0.0001–0.2 mg mL^–1^ using an isolated BAC A/A′ standard (Figure S11). Extracts from MVs, cell pellets, and cell- and MV-free supernatants were analyzed in triplicate at 0.5 mg mL^–1^ in CH_3_OH. All solvents used were of mass spectrometry grade.

### Molecular Networking and Metabolite Quantitation Workflow

Data from the untargeted LC–MS/MS were converted to mzXML format using MSConverter (version 3.0). The mzXML file was uploaded to the Global Natural Products Social (GNPS) networking server using WINSCP (version 6.1.1) to generate a molecular network.^33^ The edges were set to have a minimum cosine score of 0.7 and >6 matched fragment ions. GNPS parameters and raw molecular networks generated from the MV and cell extracts can be accessed through the links https://gnps.ucsd.edu/ProteoSAFe/status.jsp?task=d33fcc32b7994609b0bbfc8155ce2e6d and https://gnps.ucsd.edu/ProteoSAFe/status.jsp?task=b0e1f4fa82424274a1dfd6b8b5a31d41, respectively. The MS^2^ spectra were viewed in MZmine (version 3.4.16) for manual compound annotation. Targeted LC–MS/MS data were analyzed using Agilent Masshunter Workstation Qualitative Analysis (version 10.0) and Quantitative Analysis (version 10.1) software.

### Antimicrobial Testing of Membrane Vesicles

Aliquots (100 μL) of bacterial cultures were evenly spread onto a 90 × 15 mm agar plate (YP30 for marine strains and MHA for *S. aureus*) and allowed to dry at ambient temperature for 10 min. Next, 10 μL aliquots of MVs suspensions adjusted to 3.9 × 10^9^ particles/mL from different culture conditions (e.g., 24 and 48 h time points, shaking, static) were spotted on the inoculated agar surfaces. Controls consisted of 10 μL aliquots of 1× PBS, dry sterile disks loaded with 10 μg of ciprofloxacin or BAC A/A′, and air-dried sterile disks initially loaded with 10 μL of 1× PBS or CH_3_OH. Plates were incubated for 24 h at 27 °C for marine strains and 37 °C for *S. aureus*. Antibacterial activity was observed as zones with no visible bacterial growth. All tests were conducted in duplicate.

### Timeline of Antibacterial Activity

A modified MBC assay was performed to determine the timeline of antibacterial activity following treatment with the MV suspensions. In a sterile 96-well plate, 10 μL aliquots of JC3 MVs were added to 90 μL of 1× PBS and serially diluted (50 μL final volume per well). Bacterial cultures were diluted 1:1000, and 50 μL was added to wells containing MV suspension or the control. The plates were then incubated at 37 °C (*S. aureus*) or 27 °C (marine strains). Bacterial viability was assessed every 2 h (up to 8 h) by spotting a 10 μL aliquot onto a YP30 agar surface for the marine strains and Mueller–Hinton agar for *S. aureus*. YP30 and Mueller–Hinton agar plates were incubated overnight at 27 and 37 °C, respectively. Control wells were prepared using ciprofloxacin (0.1 mg mL^–1^), 1× PBS, BAC A/A′ (0.1 mg mL^–1^), and methanol. The plates were incubated at 37 or 27 °C for 24 h and imaged using a Gel Doc XR+ system (Bio-Rad).

### MIC and MBC Determinations for MVs

MIC and MBC values for *P. piscicida* JC3 MVs were determined using a microbroth dilution protocol used by the Rhode Island Infectious Diseases Research Program with some modifications.^49^ A 20 μL aliquot of JC3 MVs in PBS was transferred into a 96-well plate containing 180 μL of broth (YP30 for the *V. anguillarum* and Mueller–Hinton broth for *S. aureus*), and ten 2-fold serial dilutions were prepared. Overnight cultures of *V. anguillarum* (YP30, 27 °C, 175 rpm) and *S. aureus* (Mueller–Hinton broth, 37 °C) were diluted 1:1000 in their respective media, and 100 μL was transferred to each well. Ciprofloxacin, 1× PBS, and BAC A/A′ were used as controls. The plates were incubated in static conditions at 27 °C for *V. anguillarum* and 37 °C for *S. aureus* for 24 h and then observed for visible bacterial growth to record the MIC (lowest concentration without visible bacterial growth). For wells with no visible turbidity, 5 μL aliquots were spotted onto agar plates and incubated for 24 h. Minimum bactericidal concentrations (MBC) were designated as the lowest concentrations that did not result in colony formation.

### Large-Scale Cultivation of *P. piscicida* JC3 for Bromoalterochromides Isolation

One 10 mL of seed culture in YP30 was inoculated with the *P. piscicida* JC3 strain and incubated at 27 °C while being shaken for 24 h. One 200 mL flask containing 100 mL of YP30 media was inoculated with 1 mL from the 10 mL culture and incubated at 27 °C with shaking for 24 h. Eight 2 L flasks, each containing 1 L of YP30 media, were inoculated with 10 mL from a 100 mL culture and incubated at 27 °C with shaking (175 rpm) for the first 4 h and then statically for 8 days. The cultivation was repeated with 6 × 1 L cultures to generate additional substrate for characterization.

### Isolation of Bromoalterochromides

Lyophilized cell pellets were extracted with a dichloromethane/methanol mixture (1:1). The extract was concentrated in vacuo onto C18 resin and fractionated using reversed-phase chromatography (Combiflash Rf, Teledyne Isco) with 50 g HP C18 column (RediSep Rf, Teledyne Isco), flow rate of 20 mL/min, and water (A) and acetonitrile (B) mobile phases as follows: 10% B for 2 min, a linear gradient to 100% B over 18 min, and 100% B for 10 min. Fractions (25 mL each) collected between 16 and 19 min were combined and further fractionated by HPLC using a Shimadzu Prominence-I LC-2030C 3D HPLC equipped with a DAD unit and a XBridge Prep C18 5 μm column maintained at 40 °C and a 2 mL/min flow rate. Chromatography method using 0.1% formic acid in water (A) and 0.1% formic acid in acetonitrile (B): 50% B for 5 min, linear gradient to 75% B over 4 min, increased to 100% B over 1 min, and maintained at 100% B for 7 min. Compound yields and retention times: **1**/**2** (2 mg, *t*_R_ 10.4 min), **3**/**4** (1.8 mg, *t*_R_ 13.1 min), **5**/**6** (0.4 mg, *t*_R_ 11.9 min), and **7**/**8** (0.6 mg, *t*_R_ 14.2 min).

Bromoalterochromide E/E′ (**7**/**8**): yellow amorphous powder, UV–vis (MeOH/H_2_O 75%): λ_max_ 224 nm, 395 nm, 570 nm, 592 nm; HRMS [M + H]^+^ *m*/*z*: 884.3130 (calcd for C_41_H_55_BrN_7_O_10_^+^, 884.3188).

### Nuclear Magnetic Resonance Analysis

BAC A/A′ (**1**/**2**) and BAC E/E′ (**7**/**8**) were dissolved in deuterated dimethyl sulfoxide (DMSO-*d*_6_). Proton NMR and Correlated Spectroscopy (COSY) data were acquired for **1**/**2** by using a Bruker AVANCE III 400 MHz NMR spectrometer. Proton NMR, Correlated Spectroscopy (COSY), and HSQC data were acquired for **7**/**8** by using a Bruker AVANCE III 600 MHz NMR spectrometer.

### Marfey’s Analysis

A BAC E/E′ sample (50 μg) was heated with 6 M HCl (200 μL) at 100 °C for 4 h. The hydrolysates were dried under nitrogen at 40 °C, and the resulting residues were dissolved in Millipore water (50 μL) and 1 M NaHCO_3_ (20 μL). The resulting mixture was reacted with 1% 1-fluoro-2,4-dinitrophenyl-5-l-leucinamide (L-FDLA) in acetone (40 μL) at 37 °C for 1 h, neutralized with 1 M HCl (20 μL), and then suspended in MeCN (370 μL). An aliquot of the derivatized sample (5 μL) was injected for LC–MS analysis. In the same manner, l- and d-amino acid standards (100 μg; Thr, Val, Leu, Ile, and Asp) were individually heated with 6 M HCl (200 μL) at 100 °C for 4 h and then dried under nitrogen at 40 °C. The resulting residues were dissolved in Millipore water, derivatized by reacting with 1 M NaHCO_3_ (20 μL) and 1% FDLA in acetone (40 μL) at 37 °C for 1 h, neutralized with 1 M HCl (20 μL), and finally diluted with MeCN (370 μL) before injecting 5 μL for LC–MS analysis. Analysis was performed on a Thermo Scientific Ultimate 3000 UHPLC with a Diode Array Detection (DAD) system equipped with a Kinetex C_18_ HPLC column (150 × 4.6 mm, 5 μm) and a ThermoScientific ISQ EC mass spectrometer (ESI mass detection *m*/*z* 100–1250). Chromatography method: the mobile phase consisted of H_2_O (A), CH_3_CN (B), and 1% formic acid (C) at a flow rate of 0.6 mL/min. The mobile phase began with 15% B, linearly increased to 60% B over 55 min, and returned to 15% B for 5 min while keeping C at 5% throughout the method.

### Bioinformatic Analysis

BGCs were annotated with antiSMASH v8.^50^ For the adenylation (A) domain in the second module, antiSMASH predicted valine as the cognate substrate. By contrast, the experimentally characterized structures of bromoalterochromides E/E′ (**7**/**8**) and D/D′ (**5**/**6**) contain an isoleucine-derived residue at the corresponding position. To probe this discrepancy, we reanalyzed the same A-domain sequence with PARAS v1.0.0.^51^ PARAS ranked valine (0.992) and isoleucine (0.893) as the top candidates, indicating that this A domain may accept either substrate and thereby reconcile the sequence-based predictions with those of the Ile-containing congeners.

### Statistical Analysis and Data Visualization

Statistical analyses were done using one-way ANOVA followed by Tukey’s Honestly Significant Difference (HSD) posthoc statistical test. In all cases, mean values were compared to the other mean values. Plots were generated in GraphPad Prism (ver. 10.0.2) and R using the “ggplot2” package (version 3.4.4).

## Supplementary Material



## Notes

Additional experimental methods, figures, tables, and compound characterization data (^1^H NMR, 2D NMR, MS^2^) (PDF)

## References

[1] Gill S., Catchpole R., Forterre P. (2019). Extracellular Membrane Vesicles in
the Three Domains of Life and Beyond. FEMS Microbiol.
Rev..

[2] McMillan H. M., Kuehn M. J. (2021). The Extracellular
Vesicle Generation Paradox: A Bacterial
Point of View. EMBO J..

[3] Wang Y., Hoffmann J. P., Chou C.-W., Höner
Zu Bentrup K., Fuselier J. A., Bitoun J. P., Wimley W. C., Morici L. A. (2020). *Burkholderia thailandensis* Outer Membrane
Vesicles Exert
Antimicrobial Activity against Drug-Resistant and Competitor Microbial
Species. J. Microbiol..

[4] Bitto N. J., Chapman R., Pidot S., Costin A., Lo C., Choi J., D’Cruze T., Reynolds E. C., Dashper S. G., Turnbull L., Whitchurch C. B., Stinear T. P., Stacey K. J., Ferrero R. L. (2017). Bacterial Membrane
Vesicles Transport Their DNA Cargo
into Host Cells. Sci. Rep..

[5] Toyofuku M., Nomura N., Eberl L. (2019). Types and Origins of
Bacterial Membrane
Vesicles. Nat. Rev. Microbiol..

[6] Hagemann S., Stöger L., Kappelmann M., Hassl I., Ellinger A., Velimirov B. (2014). DNA-Bearing
Membrane Vesicles Produced by *Ahrensia
kielensis* and *Pseudoalteromonas marina*:
DNA-Bearing Membrane Vesicles. J. Basic Microbiol..

[7] Toyofuku M., Schild S., Kaparakis-Liaskos M., Eberl L. (2023). Composition and Functions
of Bacterial Membrane Vesicles. Nat. Rev. Microbiol..

[8] Alves N. J., Turner K. B., Medintz I. L., Walper S. A. (2016). Protecting Enzymatic
Function through Directed Packaging into Bacterial Outer Membrane
Vesicles. Sci. Rep..

[9] Manning A. J., Kuehn M. J. (2011). Contribution of Bacterial Outer Membrane Vesicles to
Innate Bacterial Defense. BMC Microbiol..

[10] Augustyniak D., Olszak T., Drulis-Kawa Z. (2022). Outer Membrane
Vesicles (OMVs) of *Pseudomonas aeruginosa* Provide
Passive Resistance but Not
Sensitization to LPS-Specific Phages. Viruses.

[11] Bielaszewska M., Daniel O., Nyč O., Mellmann A. (2021). In Vivo Secretion of
β-Lactamase-Carrying Outer Membrane Vesicles as a Mechanism
of β-Lactam Therapy Failure. Membranes.

[12] Yoshimura A., Saeki R., Nakada R., Tomimoto S., Jomori T., Suganuma K., Wakimoto T. (2023). Membrane-Vesicle-Mediated
Interbacterial
Communication Activates Silent Secondary Metabolite Production. Angew. Chem., Int. Ed..

[13] Biller S. J., Schubotz F., Roggensack S. E., Thompson A. W., Summons R. E., Chisholm S. W. (2014). Bacterial Vesicles in Marine Ecosystems. Science.

[14] Eze O. C., Berebon D. P., Emencheta S. C., Evurani S. A., Okorie C. N., Balcão V. M., Vila M. M. D. C. (2023). Therapeutic Potential of Marine Probiotics:
A Survey on the Anticancer and Antibacterial Effects of *Pseudoalteromonas* spp. Pharmaceuticals.

[15] Zhao W., Dao C., Karim M., Gomez-Chiarri M., Rowley D., Nelson D. R. (2016). Contributions
of Tropodithietic Acid and Biofilm Formation to the Probiotic Activity
of *Phaeobacter inhibens*. BMC
Microbiol..

[16] Offret C., Desriac F., Le Chevalier P., Mounier J., Jégou C., Fleury Y. (2016). Spotlight on Antimicrobial
Metabolites from the Marine
Bacteria *Pseudoalteromona*s: Chemodiversity and Ecological
Significance. Mar. Drugs.

[17] Parte A. C., Sardà Carbasse J., Meier-Kolthoff J. P., Reimer L. C., Göker M. (2020). List of Prokaryotic Names with Standing
in Nomenclature (LPSN) Moves to the DSMZ. Int.
J. Syst. Evol. Microbiol..

[18] Huang Y.-L., Li M., Yu Z., Qian P.-Y. (2011). Correlation between Pigmentation
and Larval Settlement Deterrence by *Pseudoalteromonas* sp. Sf57. Biofouling.

[19] Paulsen S. S., Isbrandt T., Kirkegaard M., Buijs Y., Strube M. L., Sonnenschein E. C., Larsen T. O., Gram L. (2020). Production of the Antimicrobial
Compound Tetrabromopyrrole and the Pseudomonas Quinolone System Precursor,
2-Heptyl-4-Quinolone, by a Novel Marine Species *Pseudoalteromonas
galatheae* sp. Nov. Sci. Rep..

[20] Thøgersen M. S., Delpin M. W., Melchiorsen J., Kilstrup M., Månsson M., Bunk B., Spröer C., Overmann J., Nielsen K. F., Gram L. (2016). Production of the Bioactive
Compounds Violacein and Indolmycin Is
Conditional in a maeA Mutant of *Pseudoalteromonas luteoviolacea* S4054 Lacking the Malic Enzyme. Front. Microbiol..

[21] Franks A., Haywood P., Holmström C., Egan S., Kjelleberg S., Kumar N. (2005). Isolation and Structure
Elucidation of a Novel Yellow Pigment from
the Marine Bacterium *Pseudoalteromonas tunicata*. Molecules.

[22] Wang X., Isbrandt T., Christensen E. Ø., Melchiorsen J., Larsen T. O., Zhang S.-D., Gram L. (2021). Identification and
Verification of the Prodigiosin Biosynthetic Gene Cluster (BGC) in *Pseudoalteromonas rubra* S4059. Microbiol.
Spectr..

[23] Rosario M. E., Camm J., Cavanagh D., Rowley D. C., Nelson D. R. (2021). Draft Genome
Sequence of *Pseudoalteromonas* sp. Strain JC3. Microbiol. Resour. Announce..

[24] Timmermans M. L., Picott K. J., Ucciferri L., Ross A. C. (2019). Culturing Marine
Bacteria from the Genus *Pseudoalteromonas* on a Cotton
Scaffold Alters Secondary Metabolite Production. MicrobiologyOpen.

[25] Atencio L. A., Boya P. C. A., Martin H. C., Mejía L. C., Dorrestein P. C., Gutiérrez M. (2020). Genome Mining, Microbial Interactions,
and Molecular Networking Reveals New Dibromoalterochromides from Strains
of *Pseudoalteromonas* of Coiba National Park-Panama. Mar. Drugs.

[26] Ross A. C., Gulland L. E. S., Dorrestein P. C., Moore B. S. (2015). Targeted Capture
and Heterologous Expression of the *Pseudoalteromonas* Alterochromide Gene Cluster in *Escherichia coli* Represents a Promising Natural Product Exploratory Platform. ACS Synth. Biol..

[27] Suria A. M., Tan K. C., Kerwin A. H., Gitzel L., Abini-Agbomson L., Bertenshaw J. M., Sewell J., Nyholm S. V., Balunas M. J. (2020). Hawaiian
Bobtail Squid Symbionts Inhibit Marine Bacteria via Production of
Specialized Metabolites, Including New Bromoalterochromides BAC-D/D. mSphere.

[28] Kalinovskaya N. I., Dmitrenok A. S., Kuznetsova T. A., Frolova G. M., Christen R., Laatsch H., Alexeeva Y. V., Ivanova E. P. (2008). “*Pseudoalteromonas januaria*” SUT 11 as the Source
of Rare Lipodepsipeptides. Curr. Microbiol..

[29] Speitling M., Smetanina O. F., Kuznetsova T. A., Laatsch H. (2007). Bromoalterochromides
A and A′, Unprecedented Chromopeptides from a Marine *Pseudoalteromonas maricaloris* Strain KMM 636T. J. Antibiot..

[30] Reimer S.
L., Beniac D. R., Hiebert S. L., Booth T. F., Chong P. M., Westmacott G. R., Zhanel G. G., Bay D. C. (2021). Comparative Analysis
of Outer Membrane Vesicle Isolation Methods with an *Escherichia
coli* tolA Mutant Reveals a Hypervesiculating Phenotype With
Outer-Inner Membrane Vesicle Content. Front.
Microbiol..

[31] Gnopo Y. M. D., Misra A., Hsu H.-L., DeLisa M. P., Daniel S., Putnam D. (2020). Induced Fusion and
Aggregation of Bacterial Outer Membrane
Vesicles: Experimental and Theoretical Analysis. J. Colloid Interface Sci..

[32] Medema M. H., Blin K., Cimermancic P., De Jager V., Zakrzewski P., Fischbach M. A., Weber T., Takano E., Breitling R. (2011). antiSMASH:
Rapid Identification, Annotation and Analysis of Secondary Metabolite
Biosynthesis Gene Clusters in Bacterial and Fungal Genome Sequences. Nucleic Acids Res..

[33] Wang M., Carver J. J., Phelan V. V., Sanchez L. M., Garg N., Peng Y., Nguyen D. D., Watrous J., Kapono C. A., Luzzatto-Knaan T., Porto C., Bouslimani A., Melnik A. V., Meehan M. J., Liu W.-T., Crüsemann M., Boudreau P. D., Esquenazi E., Sandoval-Calderón M., Kersten R. D., Pace L. A., Quinn R. A., Duncan K. R., Hsu C.-C., Floros D. J., Gavilan R. G., Kleigrewe K., Northen T., Dutton R. J., Parrot D., Carlson E. E., Aigle B., Michelsen C. F., Jelsbak L., Sohlenkamp C., Pevzner P., Edlund A., McLean J., Piel J., Murphy B. T., Gerwick L., Liaw C.-C., Yang Y.-L., Humpf H.-U., Maansson M., Keyzers R. A., Sims A. C., Johnson A. R., Sidebottom A. M., Sedio B. E., Klitgaard A., Larson C. B., Boya P C. A., Torres-Mendoza D., Gonzalez D. J., Silva D. B., Marques L. M., Demarque D. P., Pociute E., O’Neill E. C., Briand E., Helfrich E. J. N., Granatosky E. A., Glukhov E., Ryffel F., Houson H., Mohimani H., Kharbush J. J., Zeng Y., Vorholt J. A., Kurita K. L., Charusanti P., McPhail K. L., Nielsen K. F., Vuong L., Elfeki M., Traxler M. F., Engene N., Koyama N., Vining O. B., Baric R., Silva R. R., Mascuch S. J., Tomasi S., Jenkins S., Macherla V., Hoffman T., Agarwal V., Williams P. G., Dai J., Neupane R., Gurr J., Rodríguez A. M. C., Lamsa A., Zhang C., Dorrestein K., Duggan B. M., Almaliti J., Allard P.-M., Phapale P., Nothias L.-F., Alexandrov T., Litaudon M., Wolfender J.-L., Kyle J. E., Metz T. O., Peryea T., Nguyen D.-T., VanLeer D., Shinn P., Jadhav A., Müller R., Waters K. M., Shi W., Liu X., Zhang L., Knight R., Jensen P. R., Palsson B. Ø., Pogliano K., Linington R. G., Gutiérrez M., Lopes N. P., Gerwick W. H., Moore B. S., Dorrestein P. C., Bandeira N. (2016). Sharing and Community Curation of Mass Spectrometry
Data with Global Natural Products Social Molecular Networking. Nat. Biotechnol..

[34] Ren Y., Liu R., Zheng Y., Wang H., Meng Q., Zhu T., Yin J., Cao X., Yu Z. (2024). Biosynthetic Mechanism of the Yellow
Pigments in the Marine Bacterium *Pseudoalteromonas* sp. Strain T1lg65. Appl. Environ. Microbiol..

[35] Vijayasarathy S., Prasad P., Fremlin L. J., Ratnayake R., Salim A. A., Khalil Z., Capon R. J. (2016). C3 and
2D C3Marfey’s
Methods for Amino Acid Analysis in Natural Products. J. Nat. Prod..

[36] Nevot M., Deroncele V., Messner P., Guinea J., Mercade E. (2006). Characterization
of Outer Membrane Vesicles Released by the Psychrotolerant Bacterium *Pseudoalteromonas antarctica* NF3. Environ. Microbiol..

[37] Tebben J., Motti C., Tapiolas D., Thomas-Hall P., Harder T. (2014). A Coralline Algal-Associated Bacterium, *Pseudoalteromonas* Strain J010, Yields Five New Korormicins
and a Bromopyrrole. Mar. Drugs.

[38] Nguyen D. D., Wu C.-H., Moree W. J., Lamsa A., Medema M. H., Zhao X., Gavilan R. G., Aparicio M., Atencio L., Jackson C., Ballesteros J., Sanchez J., Watrous J. D., Phelan V. V., Van De
Wiel C., Kersten R. D., Mehnaz S., De Mot R., Shank E. A., Charusanti P., Nagarajan H., Duggan B. M., Moore B. S., Bandeira N., Palsson B. Ø., Pogliano K., Gutiérrez M., Dorrestein P. C. (2013). MS/MS Networking Guided Analysis of Molecule and Gene
Cluster Families. Proc. Natl. Acad. Sci. U.S.A..

[39] Huang W., Zhang Q., Li W., Yuan M., Zhou J., Hua L., Chen Y., Ye C., Ma Y. (2020). Development of Novel
Nanoantibiotics Using an Outer Membrane Vesicle-Based Drug Efflux
Mechanism. J. Controlled Release.

[40] Bomberger J. M., Maceachran D. P., Coutermarsh B. A., Ye S., O’Toole G. A., Stanton B. A. (2009). Long-Distance Delivery
of Bacterial Virulence Factors
by *Pseudomonas aeruginosa* Outer Membrane Vesicles. PLoS Pathog..

[41] Jäger J., Keese S., Roessle M., Steinert M., Schromm A. B. (2015). Fusion
of *Legionella pneumophila* Outer Membrane Vesicles
with Eukaryotic Membrane Systems Is a Mechanism to Deliver Pathogen
Factors to Host Cell Membranes. Cell. Microbiol..

[42] Richards G. P., Watson M. A., Needleman D. S., Uknalis J., Boyd E. F., Fay J. P. (2017). Mechanisms for *Pseudoalteromonas*
*piscicida*-induced Killing
of Vibrios and Other Bacterial
Pathogens. Appl. Environ. Microbiol..

[43] Wang F., Ghonimy A., Wang X., Zhang Y., Zhu N. (2022). Heat-killed *Pseudoalteromonas
piscicida* 2515 Decreased Bacterial Dose
and Improved Immune Resistance against *Vibrio anguillarum* in Juvenile Olive Flounder (Paralichthys olivaceus). Aquac. Res..

[44] Wang F., Ghonimy A., Wang X. (2024). Whole-Genome Sequencing of *Pseudoalteromonas piscicida* 2515 Revealed Its Antibacterial
Potency against *Vibrio anguillarum*: A Preliminary
Invitro Study. Antonie Leeuwenhoek.

[45] Caruana J. C., Walper S. A. (2020). Bacterial Membrane Vesicles as Mediators
of Microbe
– Microbe and Microbe – Host Community Interactions. Front. Microbiol..

[46] Lavoie T., Daffinee K. E., Vicent M. L., LaPlante K. L. (2025). *Staphylococcus* Biofilm Dynamics and
Antibiotic Resistance: Insights into Biofilm
Stages, Zeta Potential Dynamics, and Antibiotic Susceptibility. Microbiol. Spectr..

[47] Socha A. M., LaPlante K. L., Russell D. J., Rowley D. C. (2009). Structure–Activity
Studies of Echinomycin Antibiotics against Drug-Resistant and Biofilm-Forming *Staphylococcus aureus* and *Enterococcus faecalis*. Bioorg. Med. Chem. Lett..

[48] Chutkan, H. ; MacDonald, I. ; Manning, A. ; Kuehn, M. J. Quantitative and Qualitative Preparations of Bacterial Outer Membrane Vesicles. In Bacterial Cell Surfaces; Delcour, A. H. , Ed.; Humana Press: Totowa, NJ, 2013; Vol. 966, pp 259–272. 10.1007/978-1-62703-245-2_16 PMC4317262 23299740

[49] Performance Standards for Antimicrobial Susceptibility Testing, 34th ed.; Lewis, J. S. , Ed.; Clinical and Laboratory Standards Institute, 2024.

[50] Blin K., Shaw S., Vader L., Szenei J., Reitz Z. L., Augustijn H. E., Cediel-Becerra J. D.
D., de Crécy-Lagard V., Koetsier R. A., Williams S. E., Cruz-Morales P., Wongwas S., Segurado Luchsinger A.
E., Biermann F., Korenskaia A., Zdouc M. M., Meijer D., Terlouw B. R., van der Hooft J. J. J., Ziemert N., Helfrich E. J. N., Masschelein J., Corre C., Chevrette M. G., van Wezel G. P., Medema M. H., Weber T. (2025). antiSMASH 8.0: Extended Gene Cluster
Detection Capabilities and Analyses of Chemistry, Enzymology, and
Regulation. Nucleic Acids Res..

[51] Terlouw B. R., Huang C., Meijer D., Cediel-Becerra J. D. D., He R., Rothe M. L., Jenner M., Zhou S., Zhang Y., Fage C. D., Tsunematsu Y., van Wezel G. P., Robinson S. L., Alberti F. L., Alkhalaf L. M., Chevrette M. G., Challis G. (2025). PARAS: High-Accuracy
Machine-Learning of Substrate Specificities in Nonribosomal Peptide
Synthetases. Biorxiv.

