# Supplementary material for: Membrane Vesicle-Mediated Delivery of Antibacterial Lipopeptides by Pseudoalteromonas piscicida
Source: ACS Chem Biol. 2026 Feb 27;21(4):751–63. doi: 10.1021/acschembio.5c01016 (PMC13097078; doi:10.1021/acschembio.5c01016)
Supplement: Supplementary file 1 [file cb5c01016_si_001.pdf]

Supporting Information

Membrane Vesicle-Mediated Delivery of Antibacterial Lipopeptides by *Pseudoalteromonas piscicida*

Ololade S. Gbadebo,[1] Arvie Grace Masibag,[1,2] Margaret E. Rosario,[1] Ruolin He,[3] Yan-Song Ye,[4] Marta Gomez-Chiarri,[5] Qihao Wu,[4,*] David C. Rowley[1,*]

[1]Department of Biomedical & Pharmaceutical Sciences, College of Pharmacy, The University of Rhode Island, Kingston, RI 02881, United States

[2]Biological Sciences Department, College of Arts and Sciences, Cavite State University, Indang, Cavite, Philippines 4122

[3]Bioinformatics Group, Wageningen University, Droevendaalsesteeg 1, 6708 PB Wageningen, Netherlands.

[4]Department of Pharmaceutical Sciences, University of Pittsburgh, Pittsburgh, PA 15261, United States

[5]Department of Fisheries, Animal and Veterinary Sciences, The University of Rhode Island, Kingston, RI 02881, United States

*Corresponding emails: drowley@uri.edu; qiw153@pitt.edu

Table of Contents

Table S1: Dry weights of lyophilized cell pellets obtained from the cultures……………………………..S4

Table S2: Biosynthetic gene clusters mined from the whole genome of *P. piscicida* JC3 according to antiSMASH database……………………………………………………………………………………………………S5

Table S3: Experimental conditions of HPLC and ESI-MS/MS for the quantification of bromoalterochromides……………………………………………………………………………………………………S6

Figure S1: Growth curve for *P. piscicida* JC3…………………………………………………………………………S7

Figure S2: Bacterial cultures of *P. piscicida* JC3 grown at 27 °C in the shaken or static condition…..S8

Figure S3: Molecular networks showing the BACs in cell pellet extracts…………………………………..S9

Figure S4: Mirror plots of BAC A/A', B/B', and D/D' MS^2 experimental and literature spectra….S10

Figure S5: Isotopic fingerprints of bromoalterochromides (BACs) as shown in LC-MS spectra…S11

Figure S6: Normalized HPLC-UV chromatograms (λ =270 nm) of MV extracts and cell extracts……S12

Figure S7. LC-MS/MS networks of membrane vesicles from *P. piscicida* JC3…………………………S13

Figure S8. LC-MS/MS networks of cell pellets of *P. piscicida* JC3………………………………………S14

Figure S9: MS^2 fragmentation of BAC A/A'…………………………………………………………………S15

Figure S10: Fragmentation patterns of BAC B/B' and BAC D/D' showing the cyclic peptide chain patterns………S16

Figure S11: Standard curve for BAC A/A' quantification……………………………………………………S17

Figure S12: Targeted analysis of protonated and sodiated BAC A/A' reveal an additive response………S18

Figure S13: Retention times of BACs following targeted metabolomic analysis……………………S19

Figure S14: Comparison of BAC A/A' abundance in MV and cell pellet extracts based on peak area……S20

Figure S15: ^{1}H NMR spectrum of BAC A/A' (1/2, 400 MHz, DMSO-d_6)……………………………S21

Figure S16: COSY spectrum of BAC A/A' (1/2, 400 MHz, DMSO-d_6)…………………………………S22

Figure S17: Sensitivity of test organisms against different concentrations of *P. piscicida* JC3 MVs, ciprofloxacin, and BAC A/A'…………………………………………………………………………………………S23

Figure S18: Positive ion ESI-MS of bromoalterochromide E/E' (7/8) ……………………………………S24

Figure S19: MS/MS analysis of bromoalterochromide E/E' (7/8)……………………………………………….S25

Figure S20: MS/MS analysis of bromoalterochromide B/B' (3/4)……………………………………………...S26

Figure S21: ^{1}H NMR spectrum of bromoalterochromide E/E' (7/8, 600 MHz, DMSO-d_6)…………..S27

Figure S22: COSY spectrum of bromoalterochromide E/E' (7/8, 600 MHz, DMSO-d_6)………………S28

Figure S23: HSQC spectrum of bromoalterochromide E/E' (7/8, 600 MHz, DMSO-d_6)……………...S29

Figure S24: HMBC spectrum of *b*romoalterochromide E/E' (7/8, 600 MHz, DMSO-d_6)……………..S30

Figure S25: Key 2D NMR correlations of bromoalterochromide E/E'……………………………………..S31

Figure S26: Bioinformatics-guided stereochemical assignment of amino acids in bromoalterochromides E/E'………………………………………………………………………………………...S32

Table S1. Dry weights of lyophilized cell pellets obtained from the cultures.

Growth Condition	Dry cell weight (mg)
24 h Shaken	327.43 ± 15.03
48 h Shaken	324.47 ± 19.95
24 h Static	133.77 ± 13.25
48 h Static	181.17 ± 15.27

Data represented as mean ± SEM of three biological replicates.

Table S2. Biosynthetic gene clusters mined from the whole genome of *P. piscicida* JC3 according to antiSMASH database.

Cluster	Class	Most similar known cluster	Similarity
3.1	NRPS		
4.1	Aminopolycarboxylic acid		
5.1	NRPS		
8.1	NRPS		
11.1	NRPS	Bromoalterochromide A	85%
15.1	NRPS	Bromoalterochromide A	14%
19.1	NRPS, NRP-metallophore, T1PKS	Crochelin A	36%
20.1	NRPS		
22.1	NRPS-like		
26.1	RiPP-like		
32.1	NRPS		
35.1	NRPS	Cupriachelin	11%
37.1	NRP-metallophore		
41.1	RiPP-like		
46.1	Hydrogen-cyanide	Hydrogen Cyanide	100%
47.1	NRPS, T1PKS		
48.1	NRPS		
53.1	RiPP-like		
59.1	Thiopeptide		
60.1	NRPS, T1PKS	N-tetradecanoyl tyrosine	6%
66.1	NRPS, T1PKS	Montamide A/Capsimycin/Clifednamide A/ Frontamide A/Combamide A	22%
67.1	Crocagin		
69.1	Lanthipeptide-class-i		

Table S3. Experimental conditions of HPLC and ESI-MS/MS for the quantification of bromoalterochromides.

**Agilent 1290 Infinity II HPLC**		
**Chromatographic conditions**		
Column dimensions	150 × 4.6 mm	
Stationary phase	C18	
Particle size	2.6 µm	
Column temperature	35 °C	
Mobile phase A	Water + 0.1% Formic Acid	
Mobile phase B	Methanol + 0.1% Formic Acid	
Autosampler temperature	4 ± 1°C	
Flow rate	0.4 mL/min	
Injection volume	5 µL	
Linear gradient program		
Time (min)	A	B
0	80	20
3	80	20
13	0	100
28	0	100
30	80	20

**Agilent 6470 Triple-Quadrupole Mass Spectrometer**		
**Electrospray conditions**		
Ionization mode		ESI [+]
Capillary voltage		3500 V
Source temperature		350 °C
Sheath gas temperature		375 °C
Sheath gas flow		13 L/min
Nebulizer gas		20 psi
MS^1/MS^2 heater temperature		100 °C
Dwell time		200 ms
MS/MS		MRM
Compounds	Transitions	Collision energy
Bromoalterochromide A/A'	844.29 -> 459.2	45
	846.29 -> 459.2	45
	866.29 -> 753.2	21
	868.29 -> 755.2	25
Bromoalterochromide B/B'	870.29 -> 459.2	45
	872.29 -> 459.2	45
	892.29 -> 779.2	21
	894.29 -> 781.2	25
Bromoalterochromide D/D'	858.29 -> 473.2	45
	860.29 -> 473.2	45
	880.29 -> 767.2	21
	882.29 -> 769.2	25
Bromoalterochromide E/E'	884.29 -> 473.2	45
	886.29 -> 473.2	45
	906.29 -> 793.2	21
	908.29 -> 795.2	25

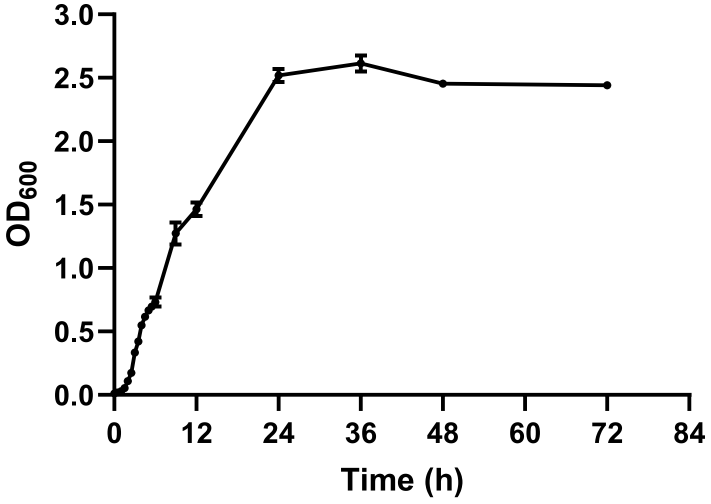

Figure S1. Growth curve for *P. piscicida* JC3. A 24 h bacterial inoculum was introduced into 100 mL of YP30 media. Culture was grown at 27 °C with shaking at 175 rpm, and aliquots were taken at intervals for absorbance measurements at 600 nm.

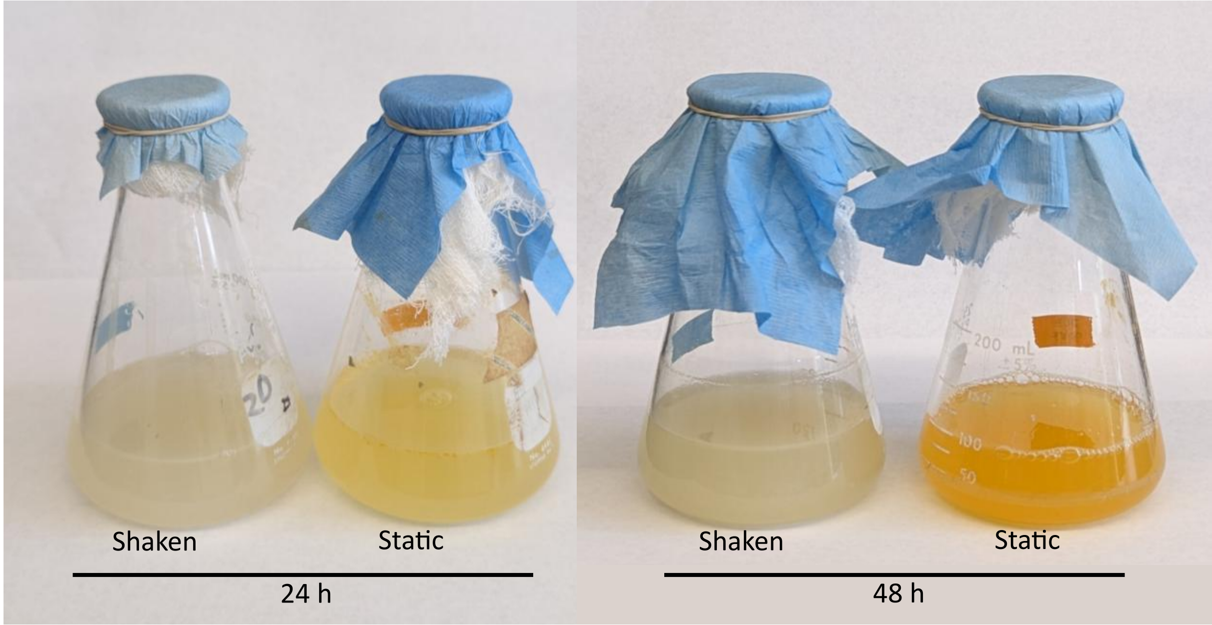

Figure S2. Bacterial cultures of *P. piscicida* JC3 grown at 27 °C in the shaken (175 rpm) or static condition.

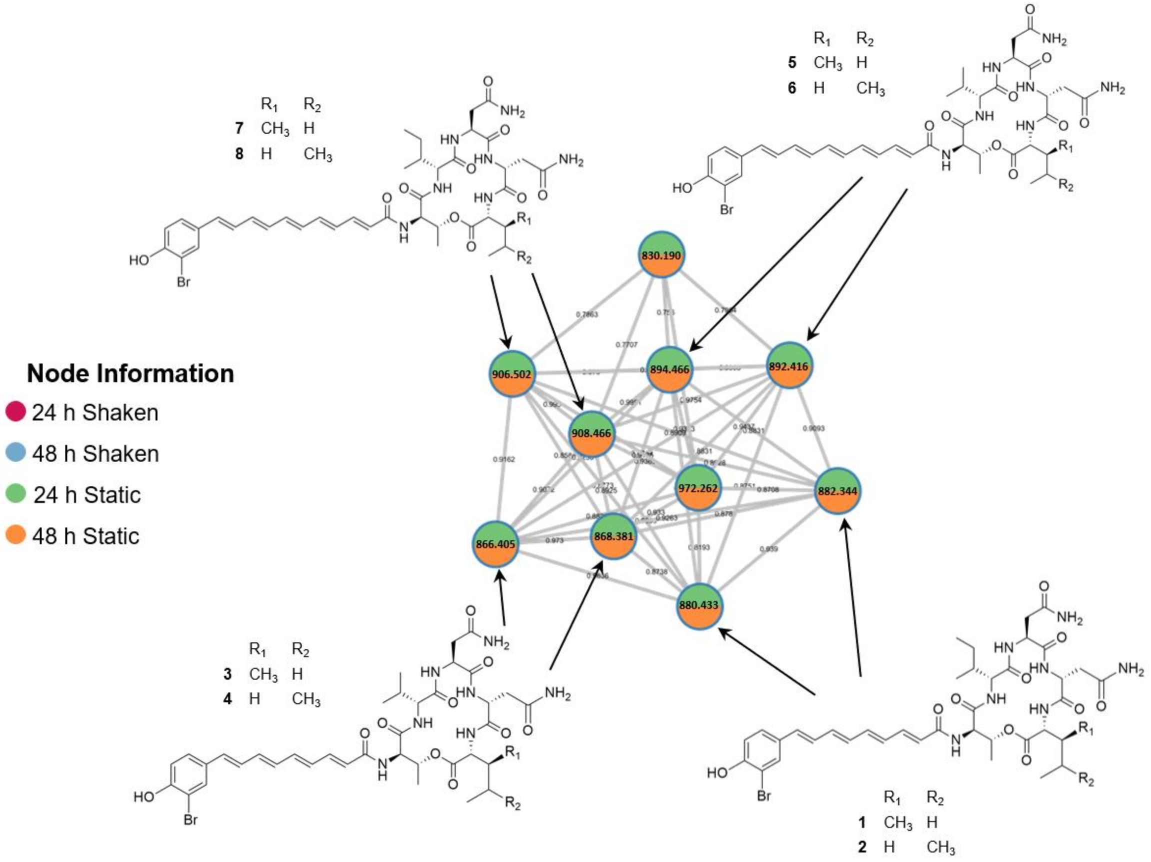

Figure S3. Molecular networks showing the BACs in cell pellet extracts. Arrows link the compounds represented by the nodes to their respective chemical structures. Pie charts in the nodes indicate the relative abundance of the *m/z* of the compounds in the different cell pellet extracts.

Bromoalterochromide A/A' [M+H]+

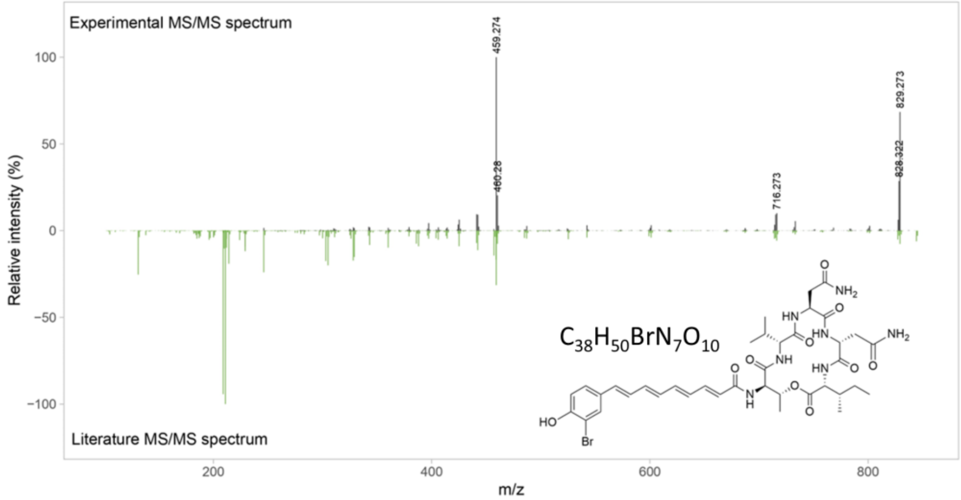

Bromoalterochromide B/B' [M+H]+

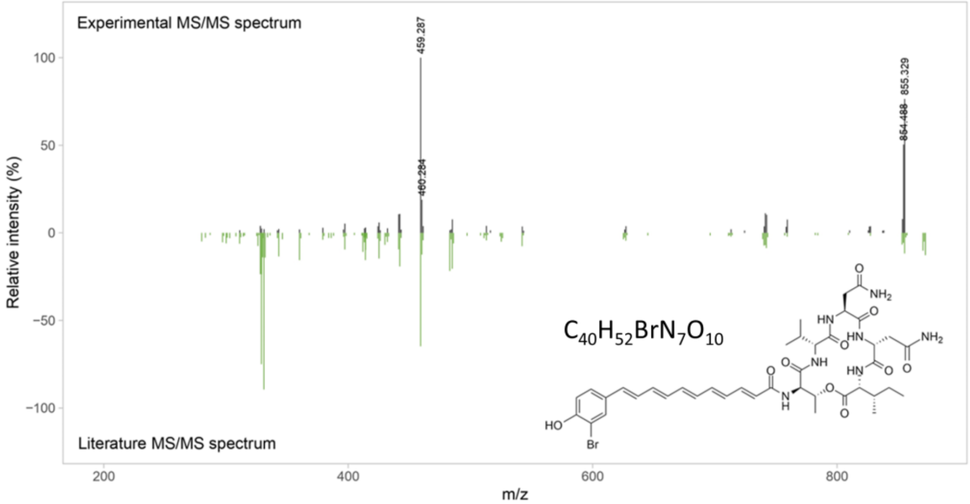

Bromoalterochromide D/D' [M+H]+

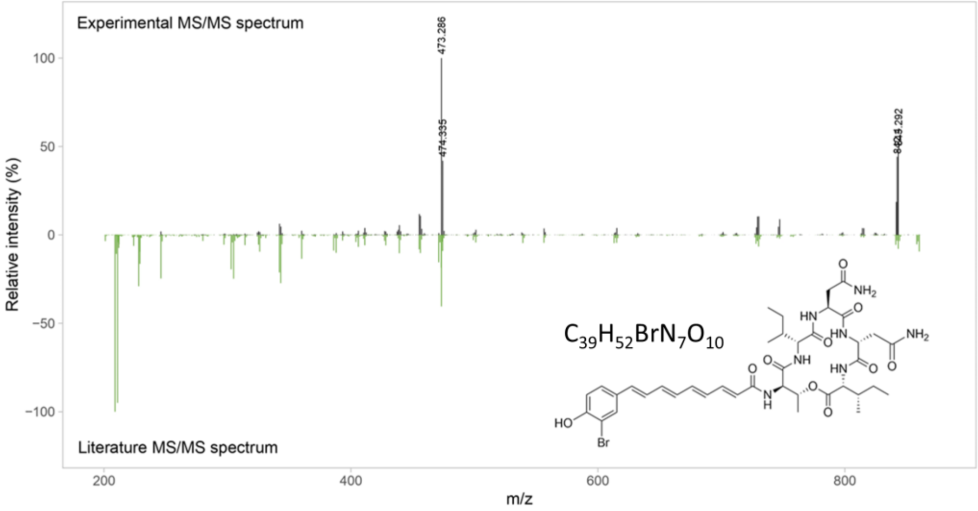

Figure S4. Mirror plots of BAC A/A', B/B', and D/D' MS^2 experimental and literature spectra.[2,3]

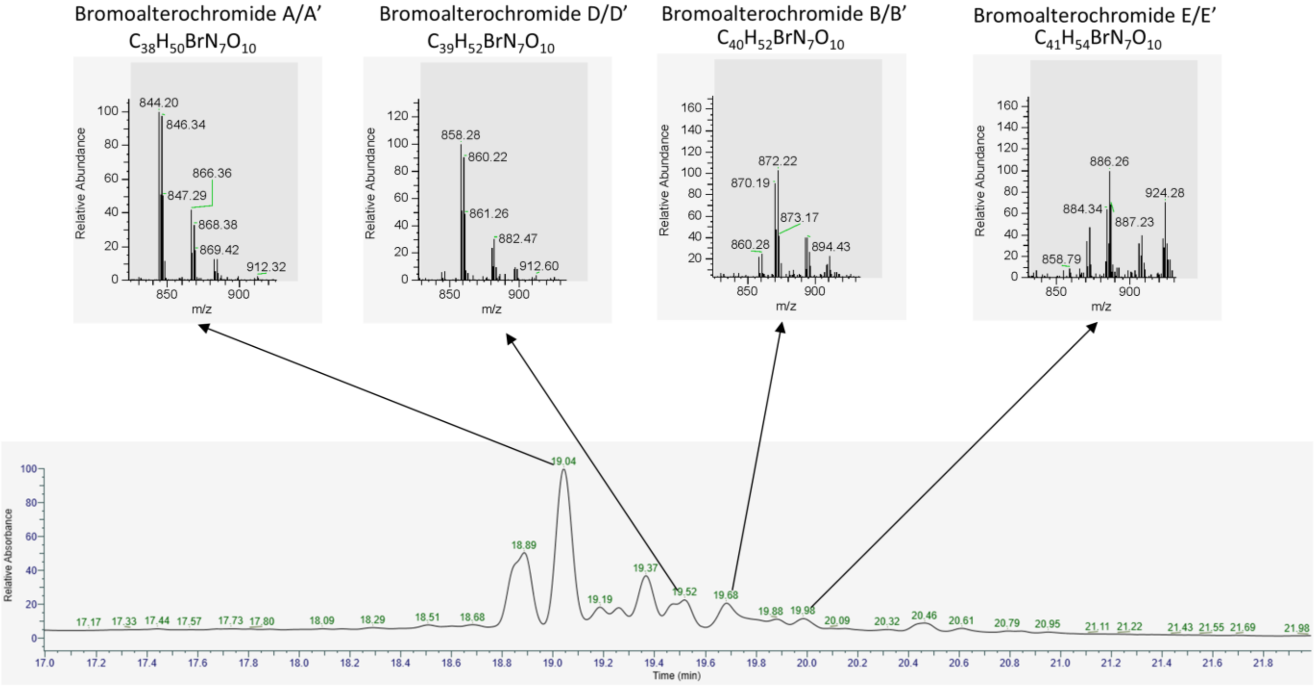

Figure S5. Isotopic fingerprints of bromoalterochromides (BACs) are shown in LC-MS spectra.

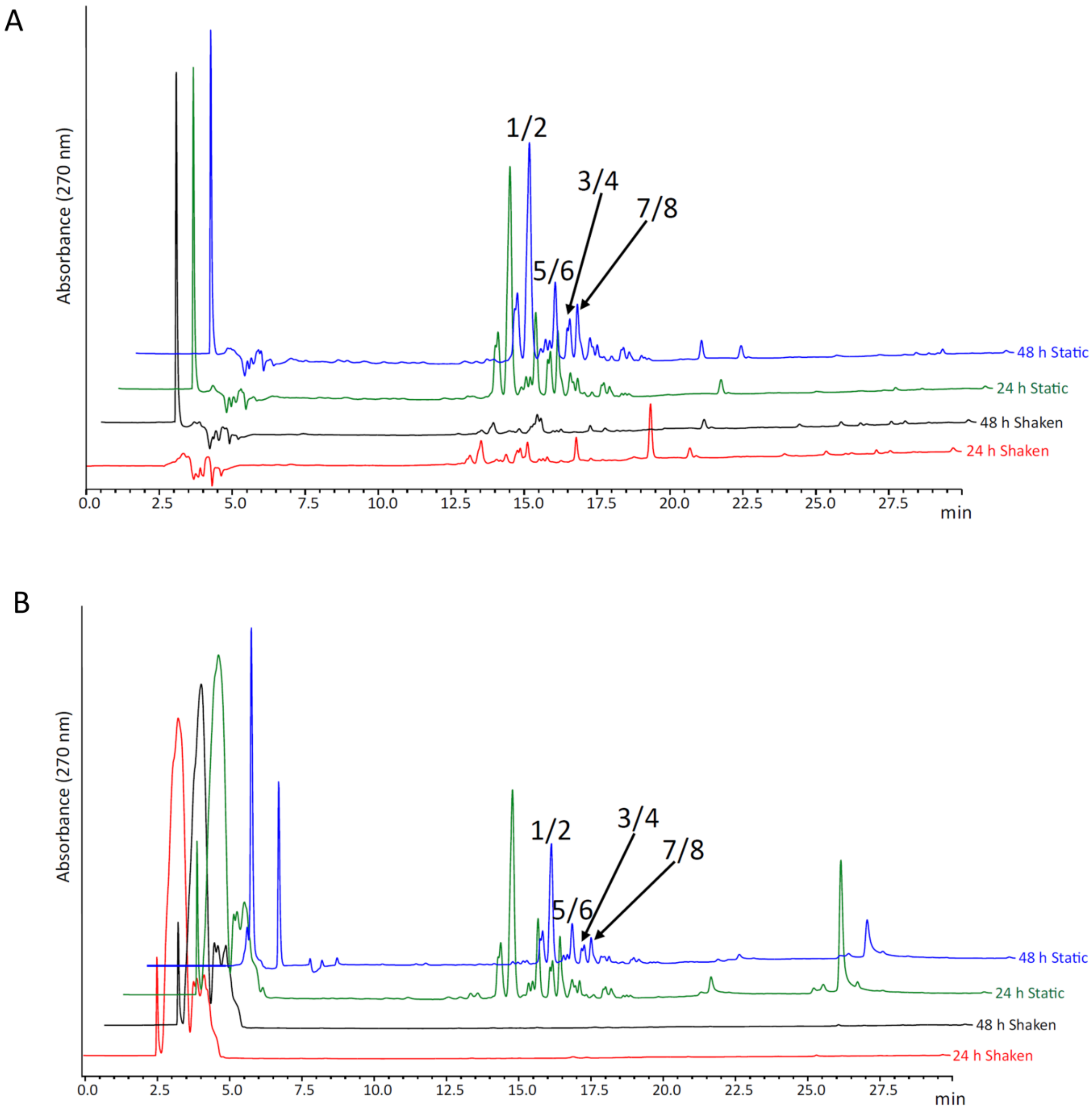

Figure S6. Normalized HPLC-UV chromatograms (λ =270 nm) of (A) MV extracts and (B) cell extracts.

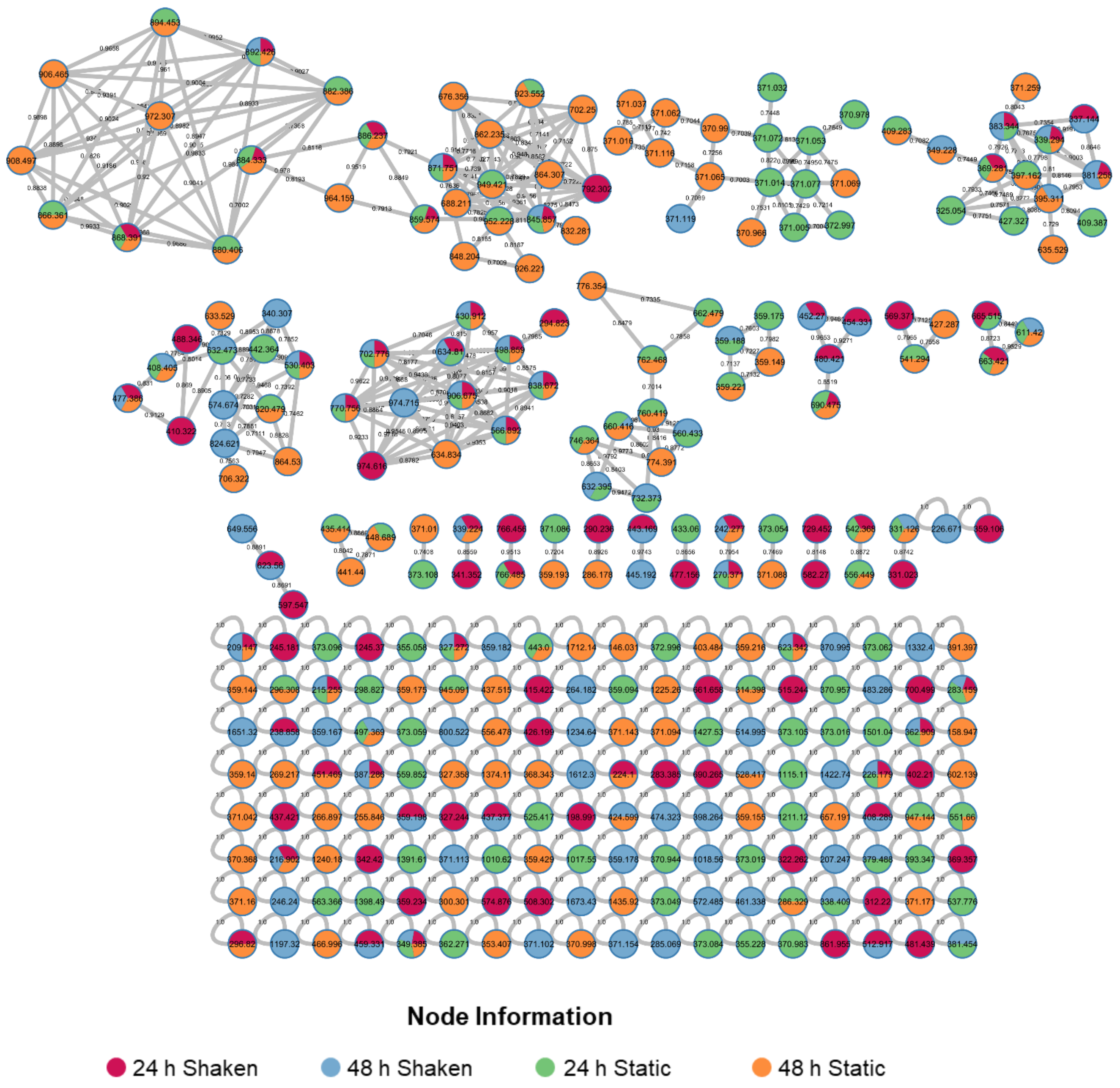

Figure S7. LC-MS/MS networks of membrane vesicles from *P. piscicida* JC3. Pie slices indicate the relative abundance of metabolites in extracts from the different growth conditions.

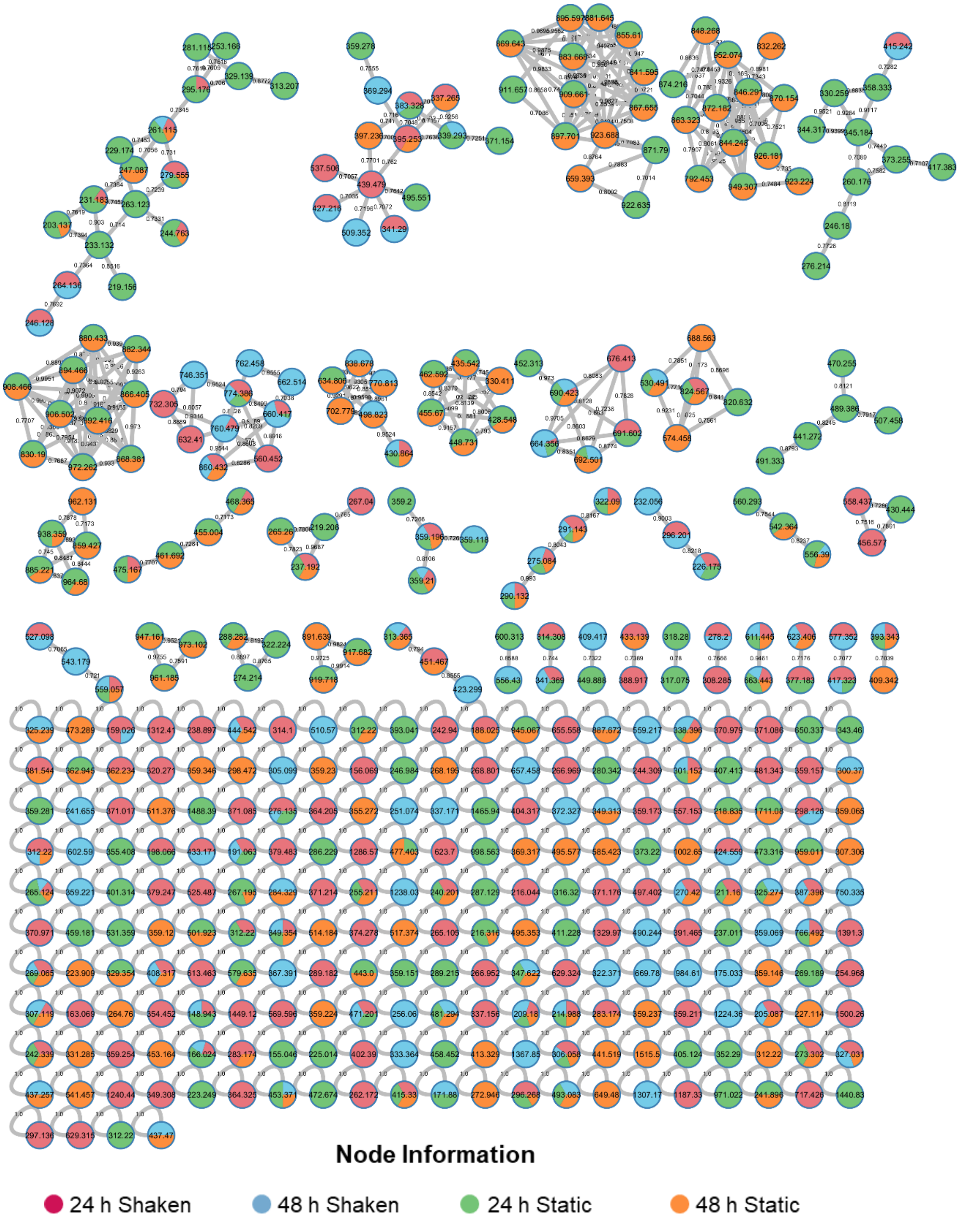

Figure S8. LC-MS/MS networks of cell pellets of *P. piscicida* JC3. Pie slices indicate the relative abundance of metabolites in extracts from the different growth conditions.

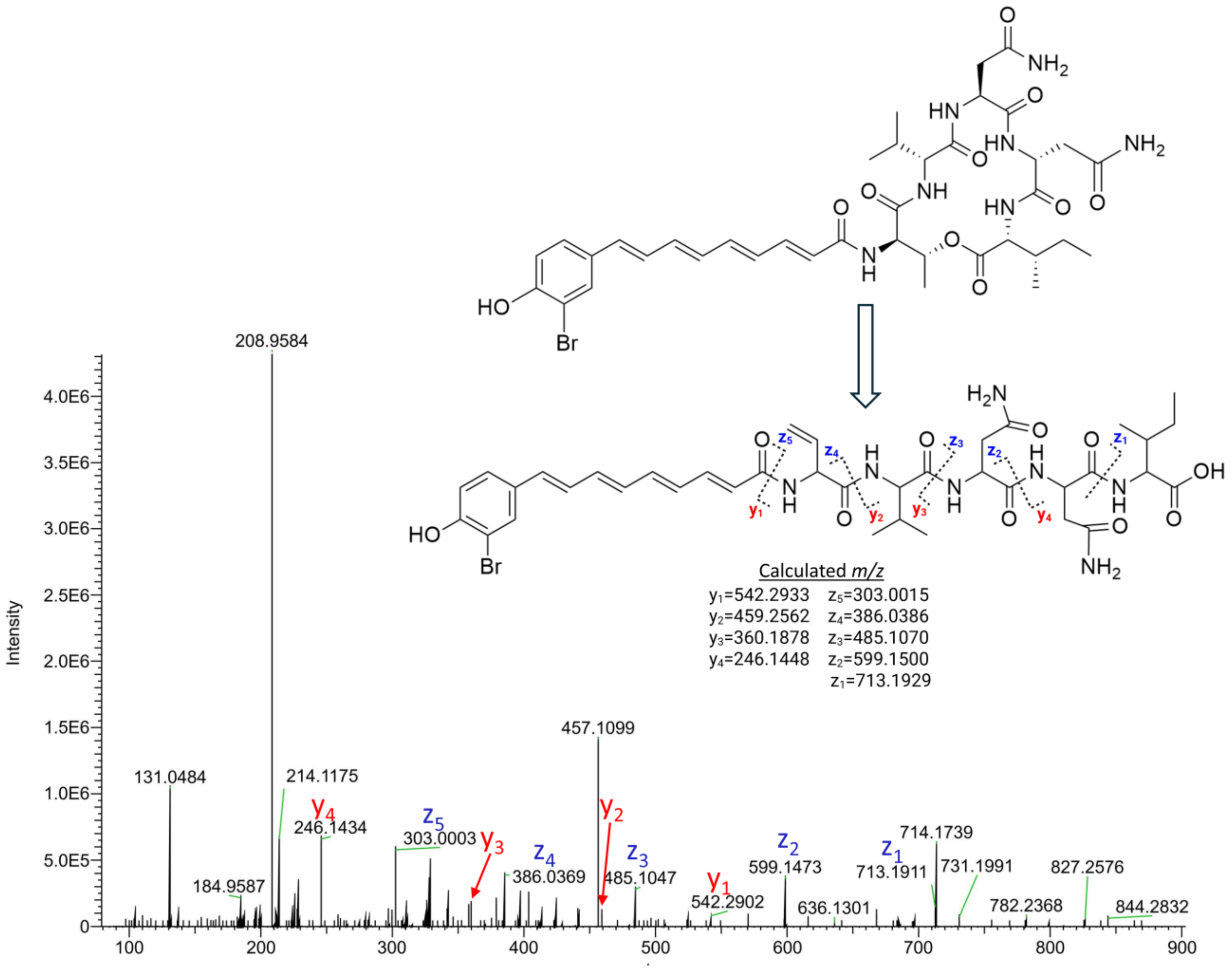

Figure S9. MS^2 fragmentation of BAC A/A'.[1]

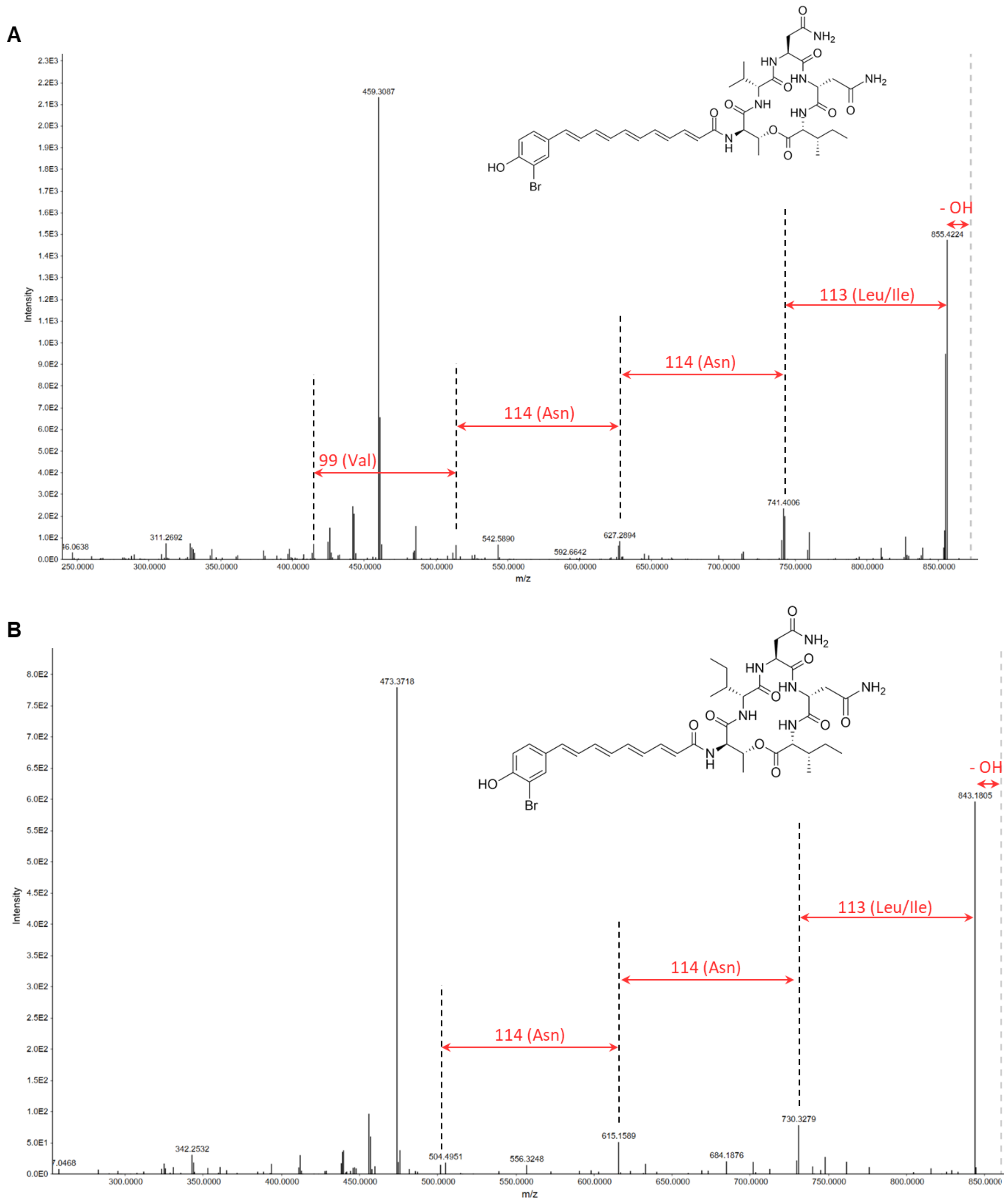

Figure S10. Fragmentation patterns of (A) BAC B/B' and (B) BAC D/D' showing the cyclic peptide chain patterns.

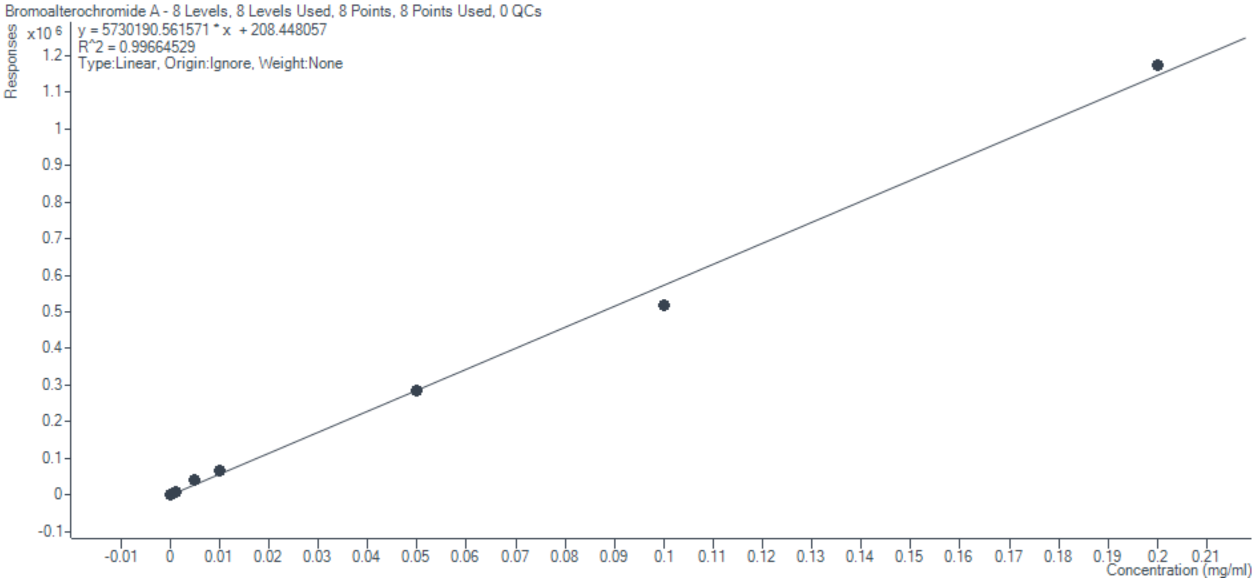

Figure S11. Standard curve for BAC A/A' quantification. BAC A/A' standards in the concentration range 0.0001 – 0.2 mg/mL were analyzed using targeted reverse-phase LC-MS/MS methods. MS data was acquired in the positive ion and multiple Reaction Monitoring (MRM) modes with *m/z* transitions of 844.29 -> 459.2, 846.29 -> 459.2, 866.29 -> 753.2 and 868.29 -> 755.2.

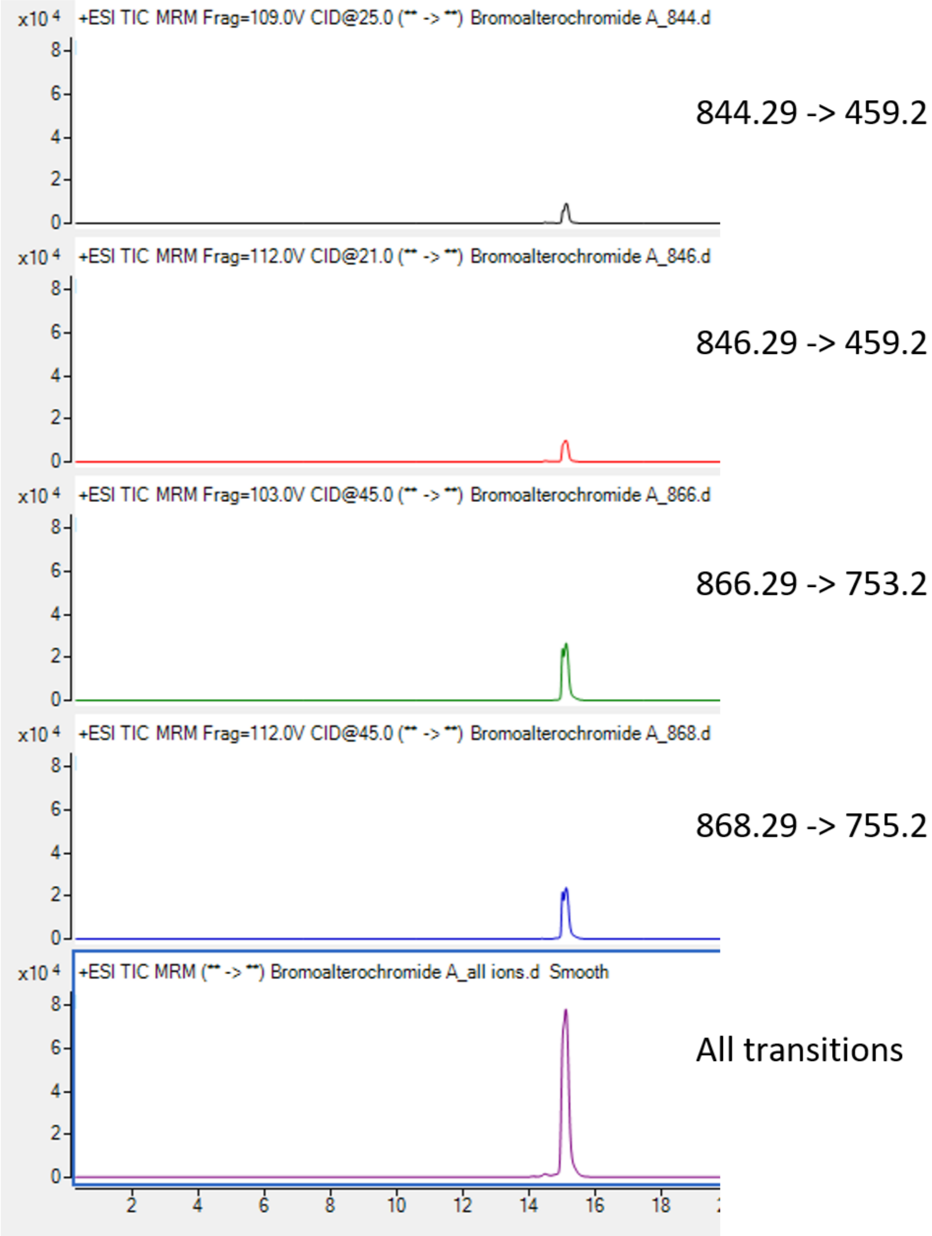

Figure S12. Targeted analysis of protonated and sodiated BAC A/A' reveals an additive response. The parent–product *m/z* transitions for BAC A/A' were individually targeted using a triple quadrupole MS. All the transitions were incorporated into a single method. This showed an additive response and was adopted in the downstream analysis of BACs in *P. piscicida* JC3 MV and cell extracts.

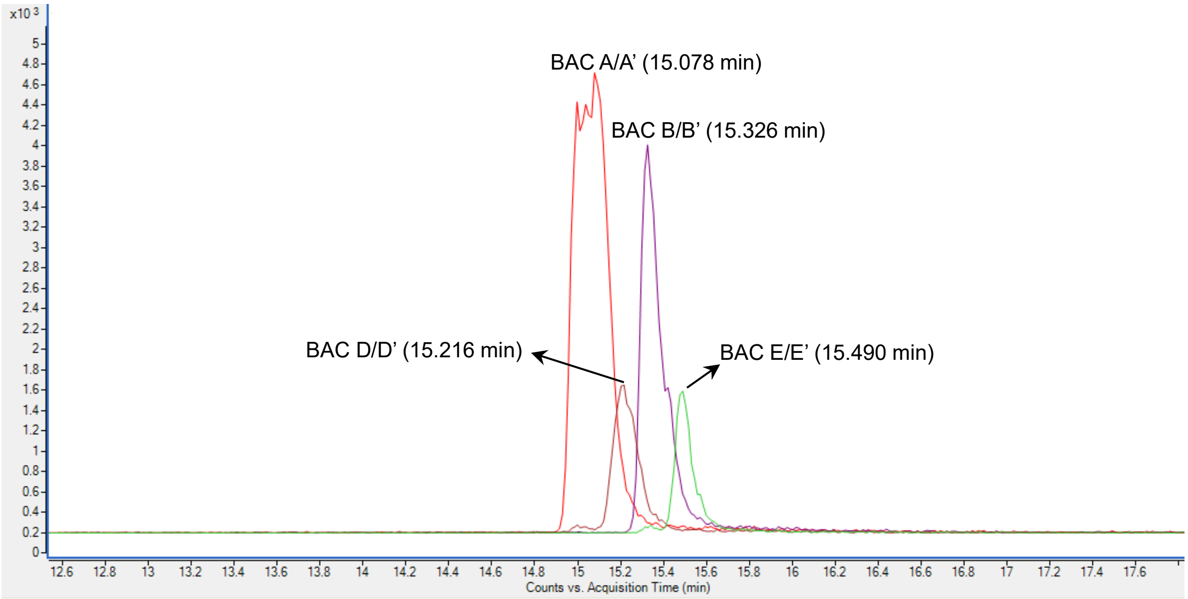

Figure S13. Retention times of BACs following targeted metabolomic analysis. Targeted LC/MS/MS analysis of BACs in JC3 MV and cell extracts reveals close retention times of BACs, suggesting structural similarities.

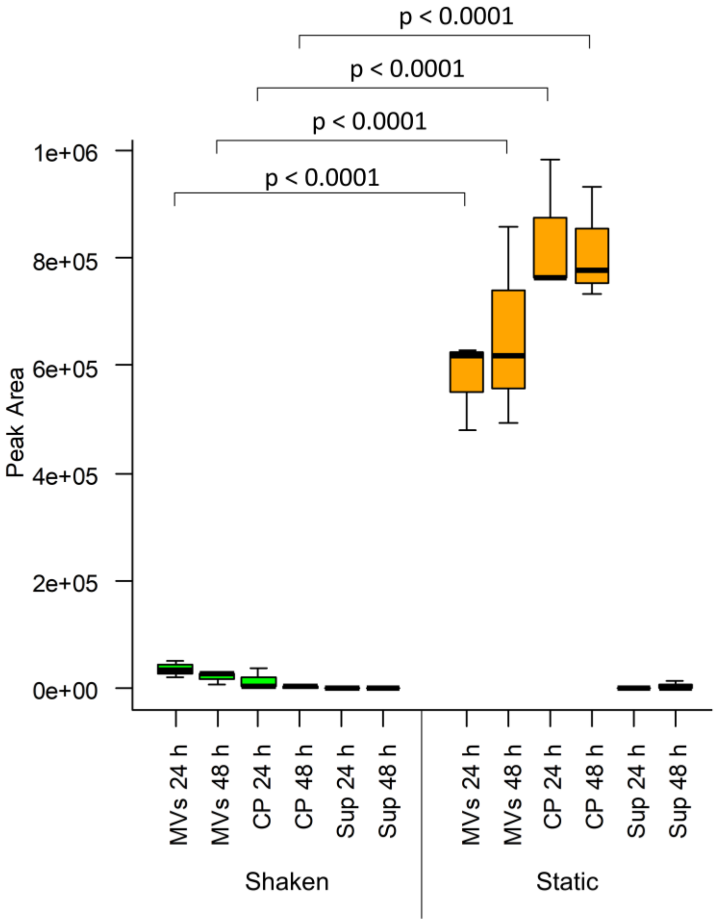

Figure S14. Comparison of BAC A/A' abundance in MV and cell pellet extracts based on peak area. The boxes show the interquartile range, while the black lines within the boxes are the median values from three biological replicates. The whiskers show the maximum and minimum values. Statistics are by one-way analysis of variance (ANOVA) followed by Tukey's correction. MVs = membrane vesicle extracts, CP = cell pellet extracts, Sup = cell- and MV-free supernatant extracts.

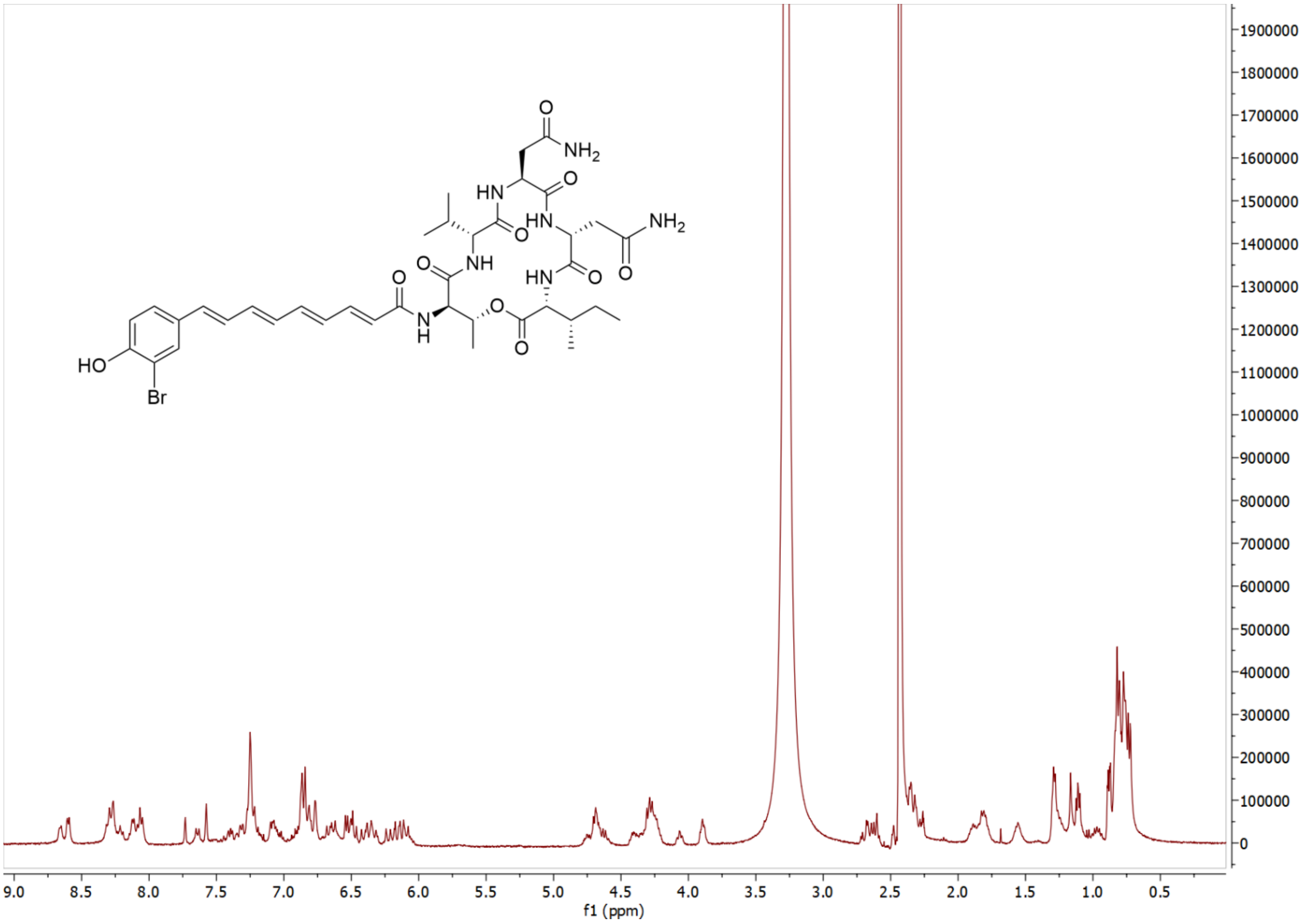

Figure S15. ^{1}H NMR analysis of BAC A/A' (**1**/**2**, 400 MHz, DMSO-d_6).

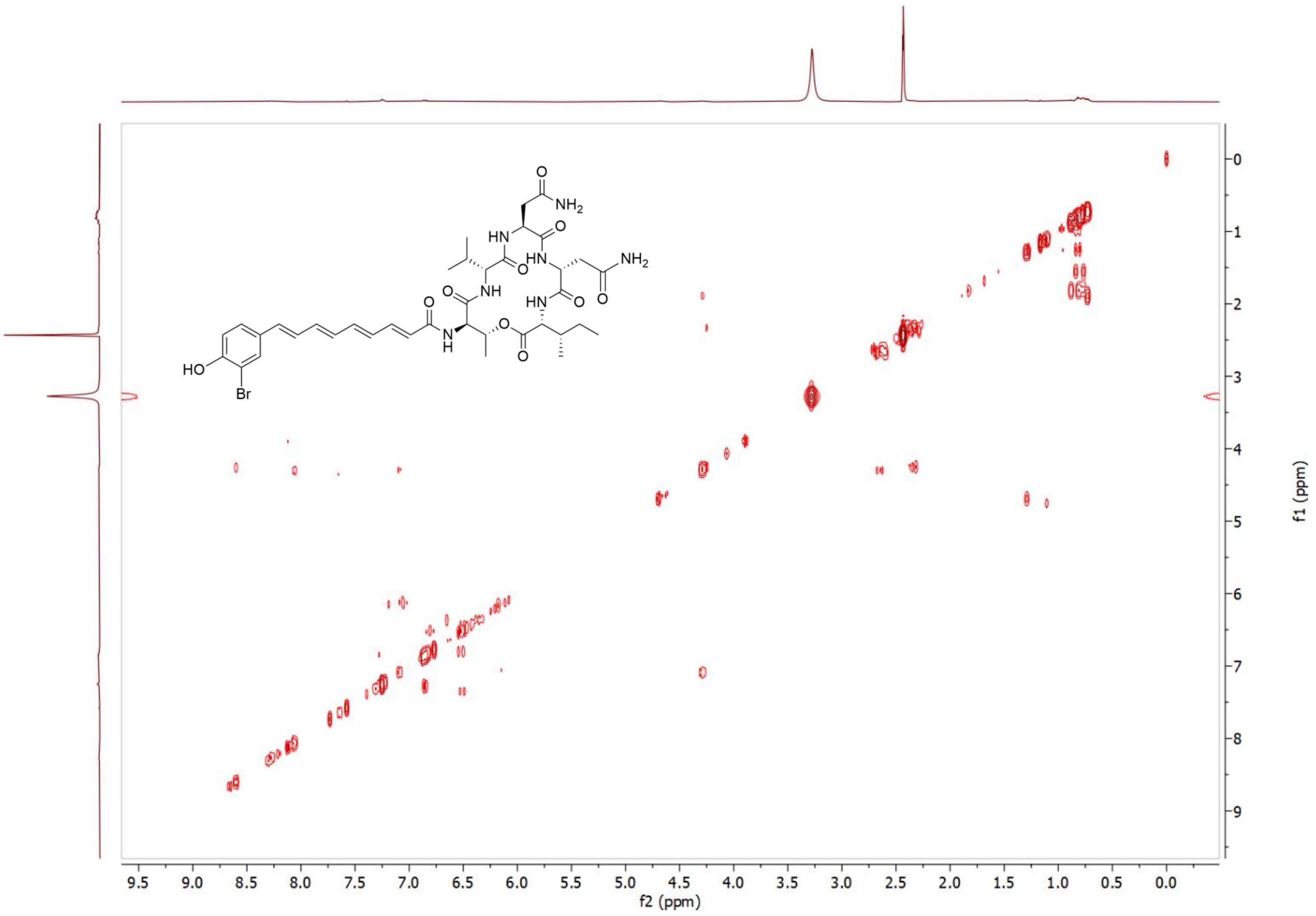

Figure S16. COSY spectrum of BAC A/A' (**1**/**2**, 400 MHz, DMSO-d_6).

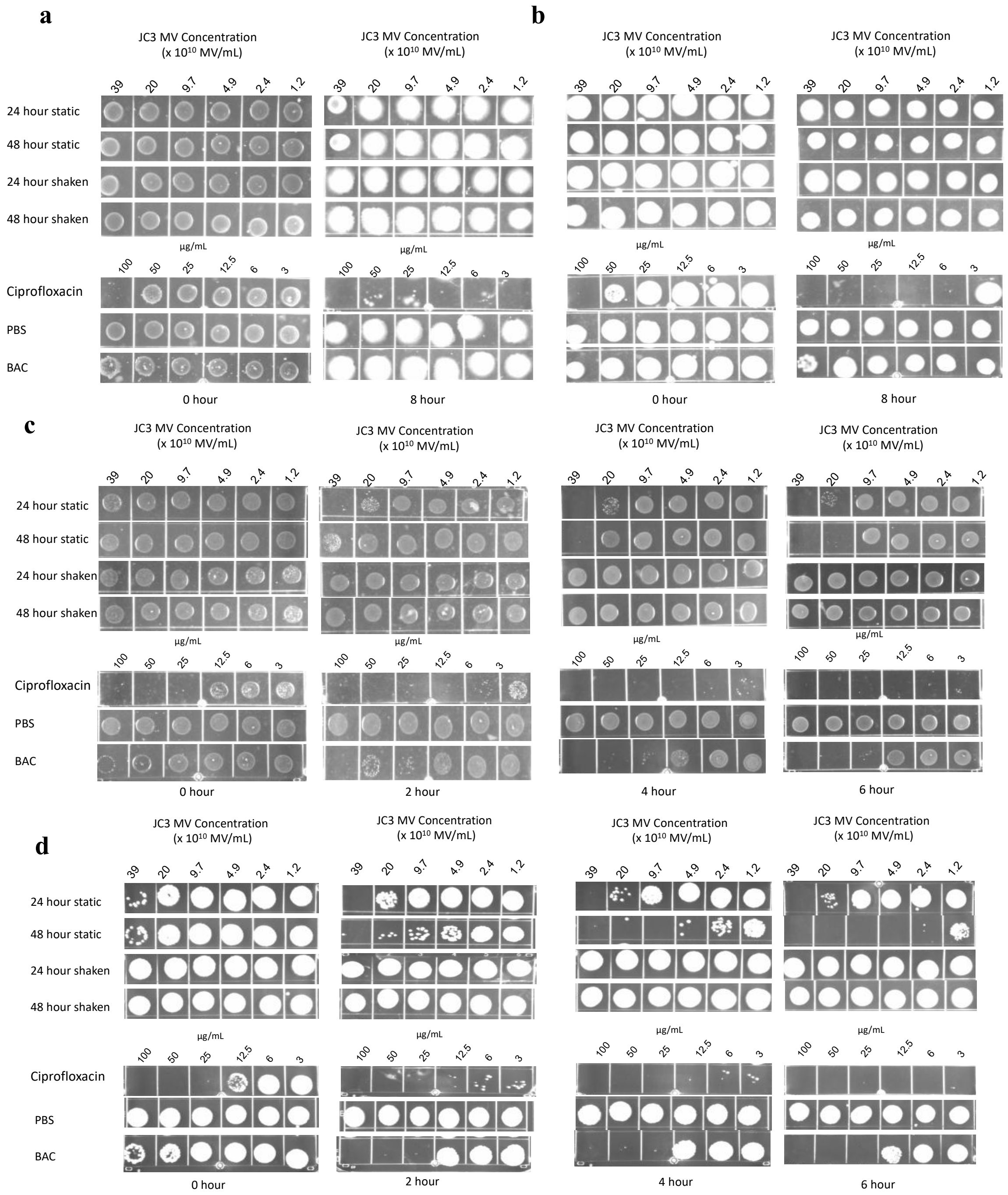

Figure S17. Sensitivity of test organisms against different concentrations of *P. piscicida* JC3 MVs, ciprofloxacin, and BAC A/A'. Qualitative assessment of cell viability by spot assays (**a**) *V. parahaemolyticus PSU5579*, (**b**) V. *coralliilyticus* RE22, (**c**) *V. anguillarum* NB10, and (**d**) *S. aureus* DMS 1104.

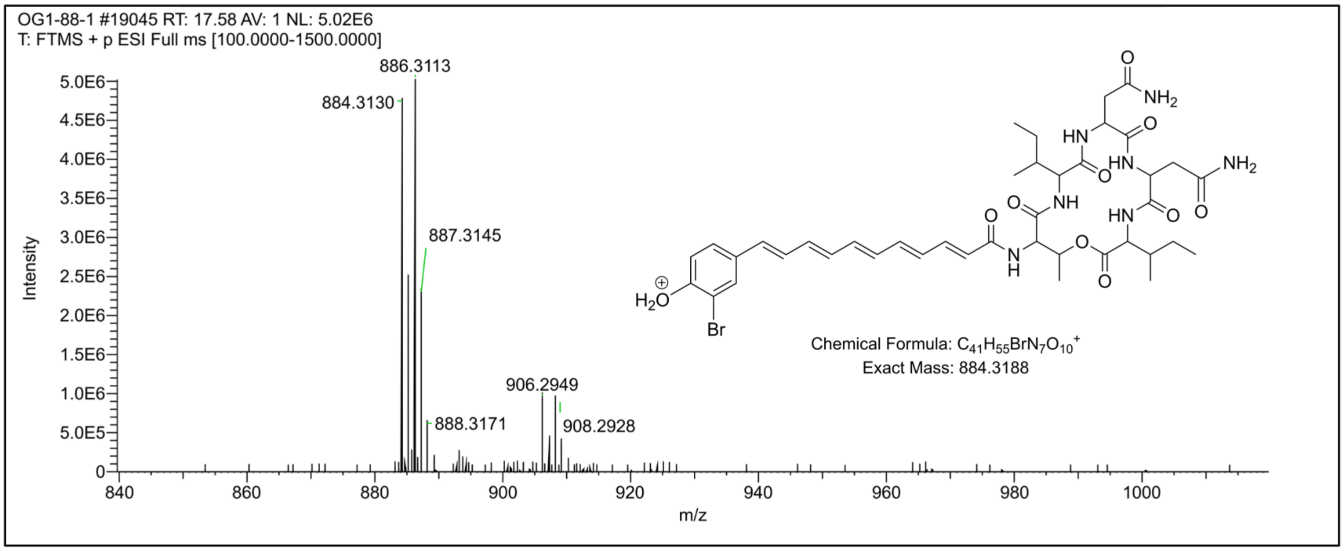

Figure S18. Positive ion ESI-MS of bromoalterochromide E/E' (**7**/**8**), $[M+H]^+$, m/z_{calcd} 884.3188, m/z_{obs} 884.3130.

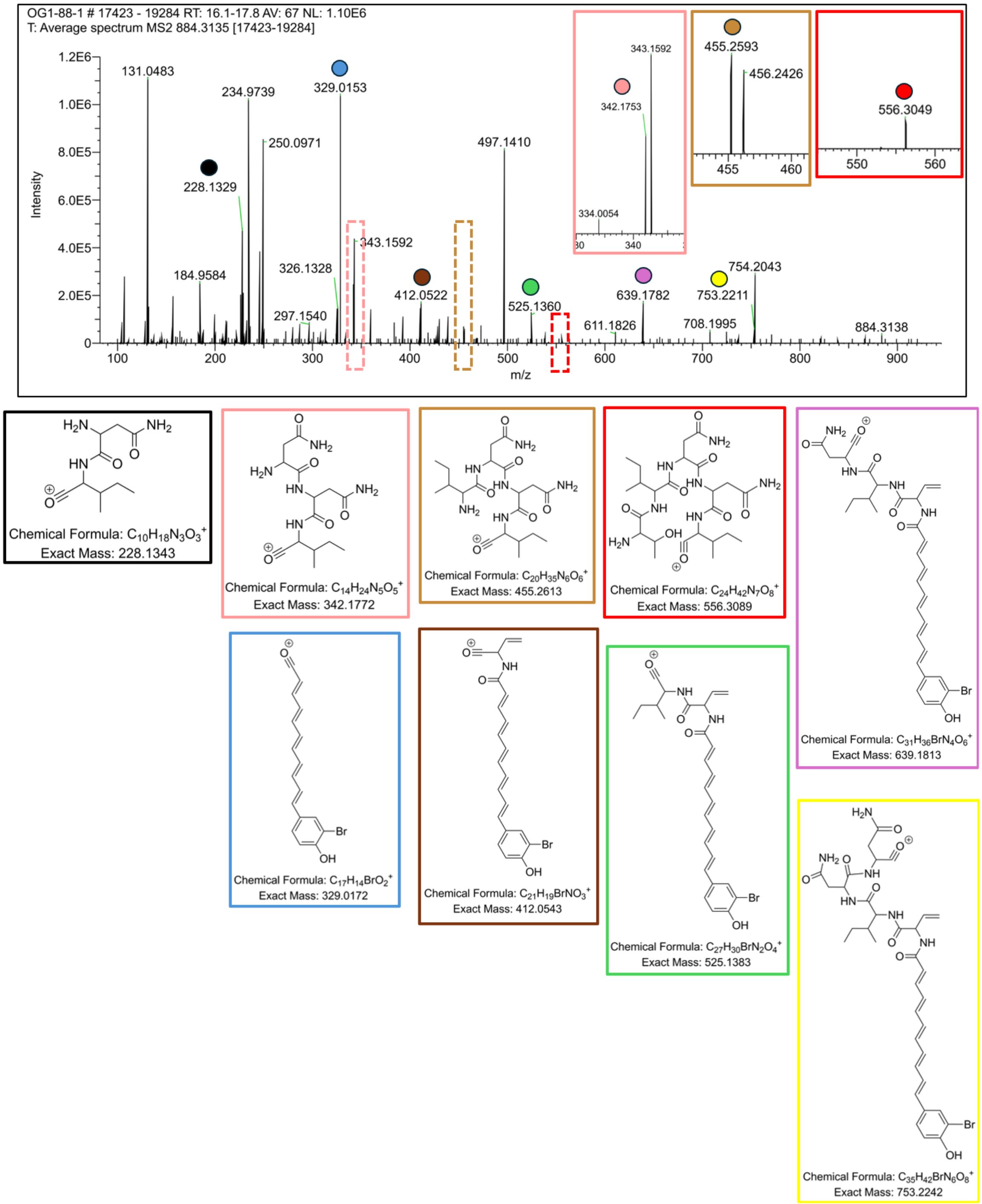

Figure S19. MS/MS analysis of bromoalterochromide E/E' (**7**/**8**). Observed MS/MS fragments include; A: $C_{10}H_{18}N_3O_3^+$, $[M+H]^+$, m/z_{calcd} 228.1343, m/z_{obs} 228.1329; B: $C_{14}H_{24}N_5O_5^+$, $[M+H]^+$, m/z_{calcd} 342.1772, m/z_{obs} 342.1753; C: $C_{17}H_{14}BrO_2^+$, $[M+H]^+$, m/z_{calcd} 329.0172, m/z_{obs} 329.0153; D: $C_{20}H_{35}N_6O_6^+$, $[M+H]^+$, m/z_{calcd} 455.2613, m/z_{obs} 455.2593; E: $C_{21}H_{19}BrNO_3^+$, $[M+H]^+$, m/z_{calcd} 412.0543, m/z_{obs} 412.0522; F: $C_{24}H_{42}N_7O_8^+$, $[M+H]^+$, m/z_{calcd} 556.3089, m/z_{obs} 556.3049; G: $C_{27}H_{30}BrN_2O_4^+$, $[M+H]^+$, m/z_{calcd} 525.1383, m/z_{obs} 525.1360; H: $C_{31}H_{36}BrN_4O_6^+$, $[M+H]^+$, m/z_{calcd} 639.1813, m/z_{obs} 639.1782; I: $C_{35}H_{42}BrN_6O_8^+$, $[M+H]^+$, m/z_{calcd} 753.2242, m/z_{obs} 753.2211.

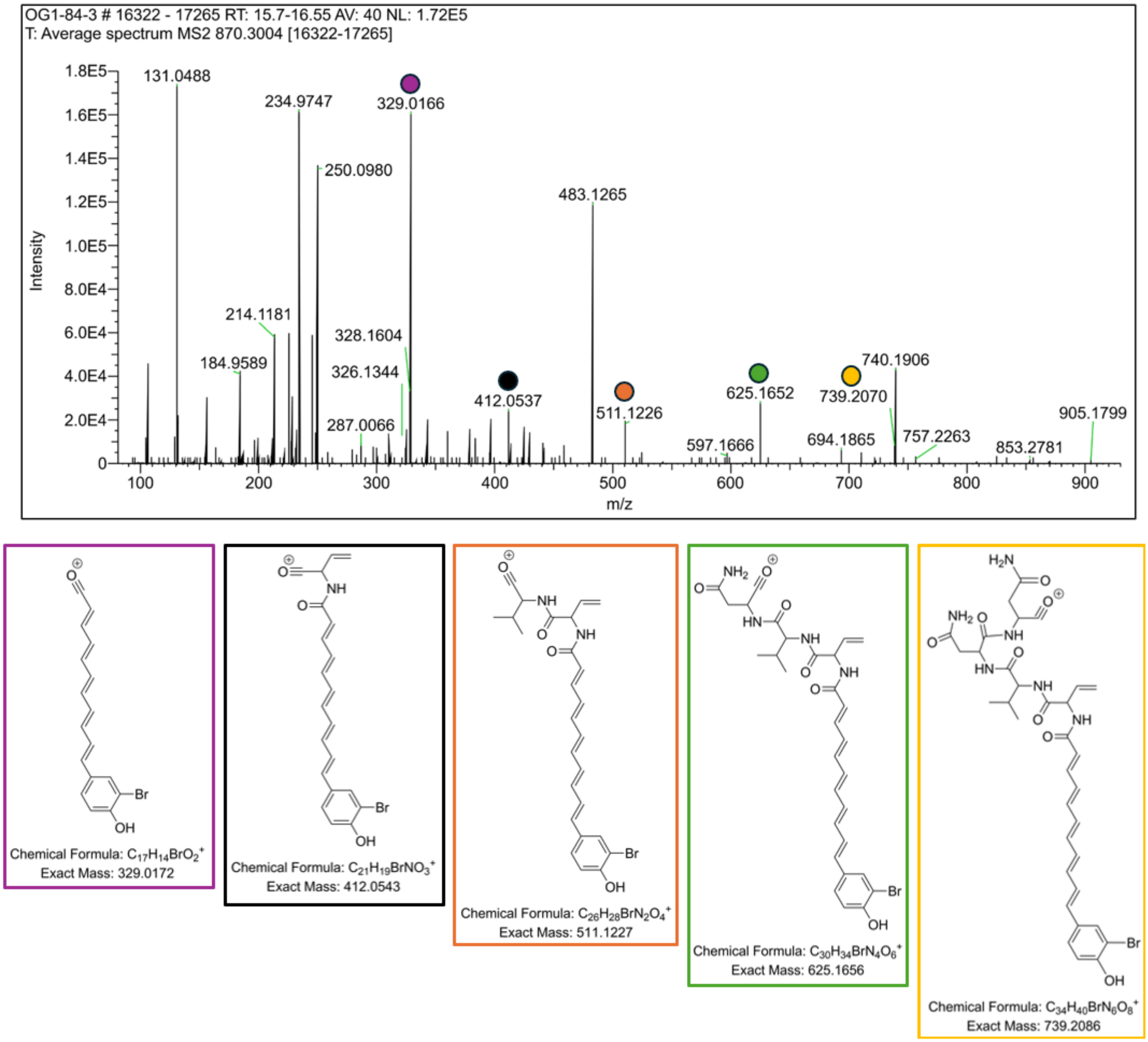

Figure S20. MS/MS analysis of Bromoalterochromide B/B' (**3**/**4**). Observed MS/MS fragments include; A: $C_{17}H_{14}BrO_2^+$, $[M+H]^+$, m/z_{calcd} 329.0172, m/z_{obs} 329.0166; B: $C_{21}H_{19}BrNO_3^+$, $[M+H]^+$, m/z_{calcd} 412.0543, m/z_{obs} 412.0537; C: $C_{26}H_{28}BrN_2O_4^+$, $[M+H]^+$, m/z_{calcd} 511.1227, m/z_{obs} 511.1226; D: $C_{30}H_{34}BrN_4O_6^+$, $[M+H]^+$, m/z_{calcd} 625.1656, m/z_{obs} 625.1652; E: $C_{34}H_{40}BrN_6O_8^+$, $[M+H]^+$, m/z_{calcd} 739.2086, m/z_{obs} 739.2070.

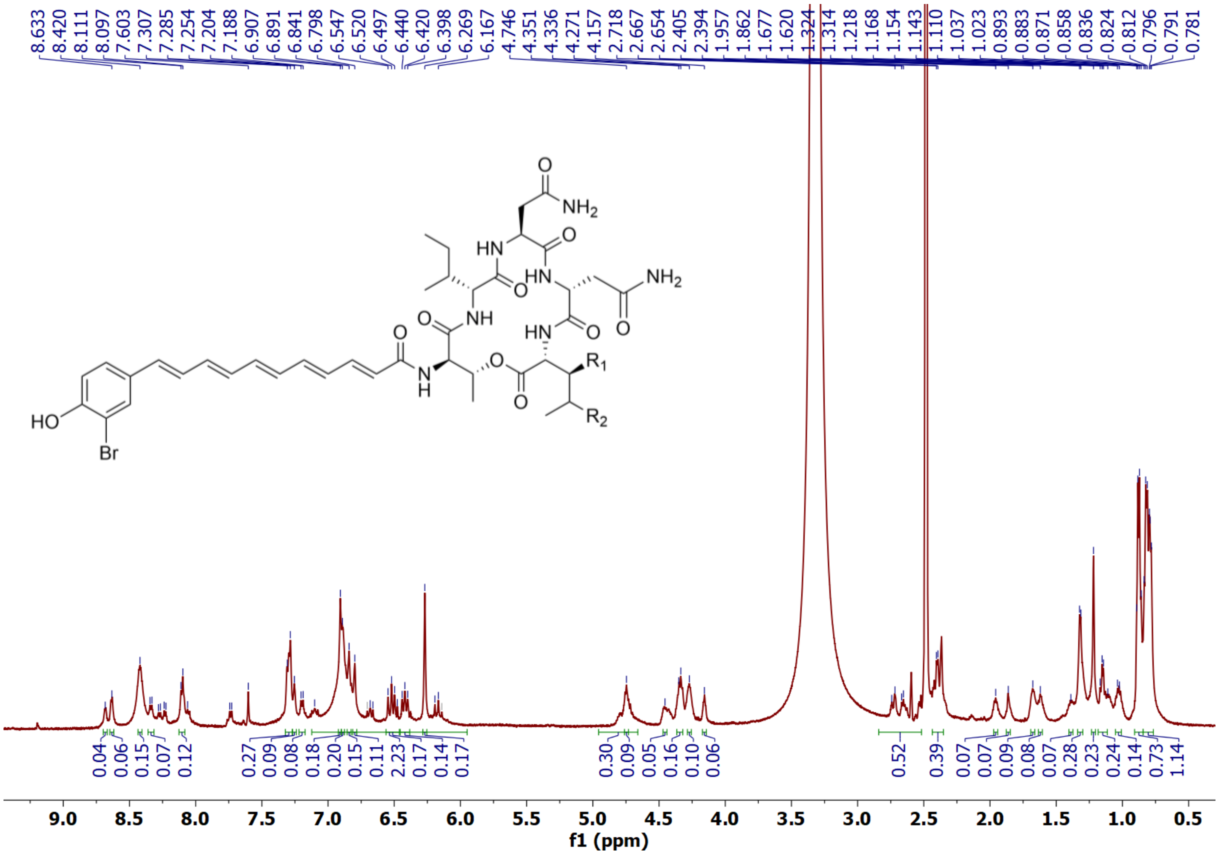

Figure S21. ^{1}H NMR spectrum of Bromoalterochromide E/E' (**7**/**8**, 600 MHz, DMSO-d_6)

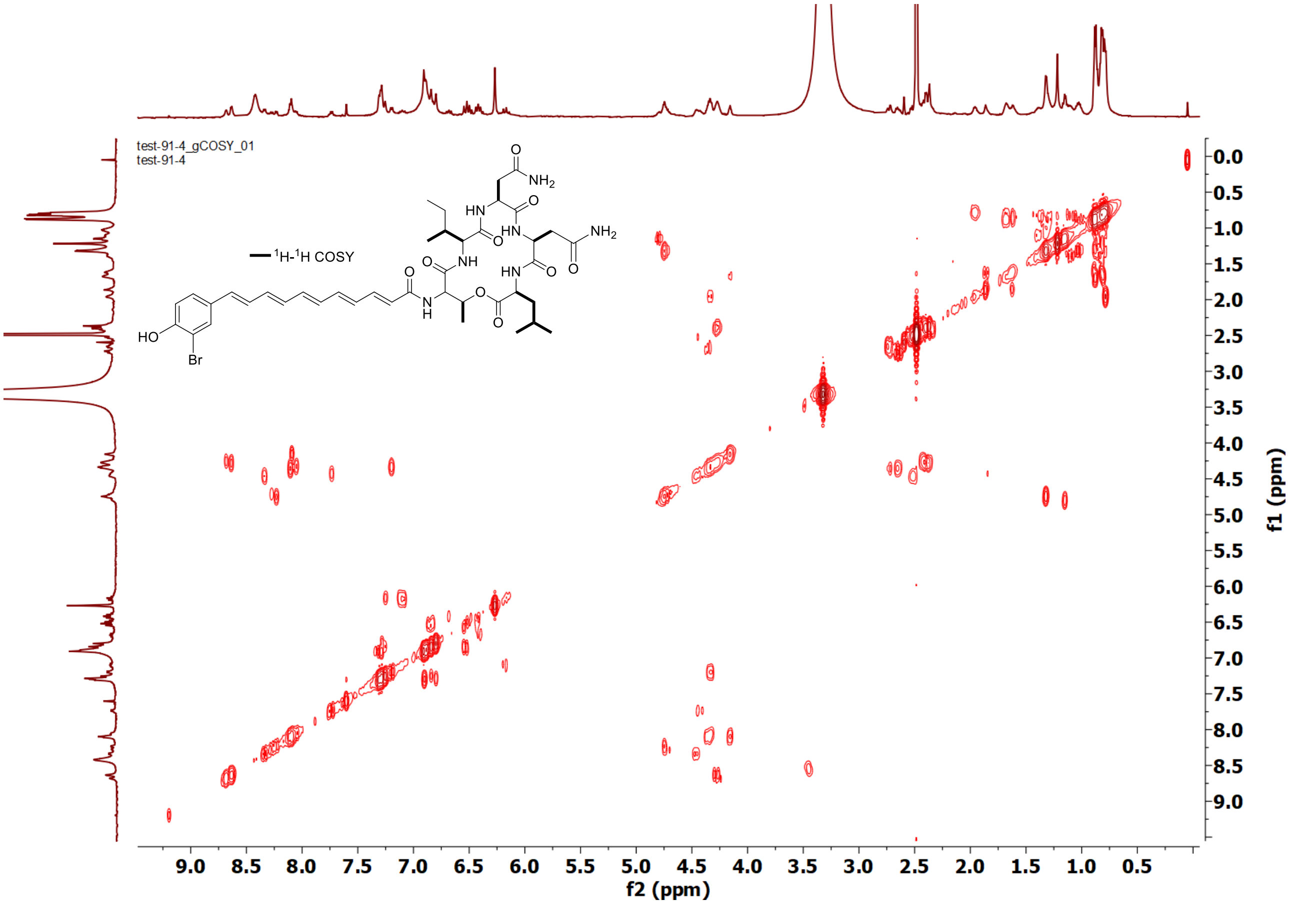

Figure S22. COSY spectrum of Bromoalterochromide E/E' (**7**/**8**, 600 MHz, DMSO-d_6).

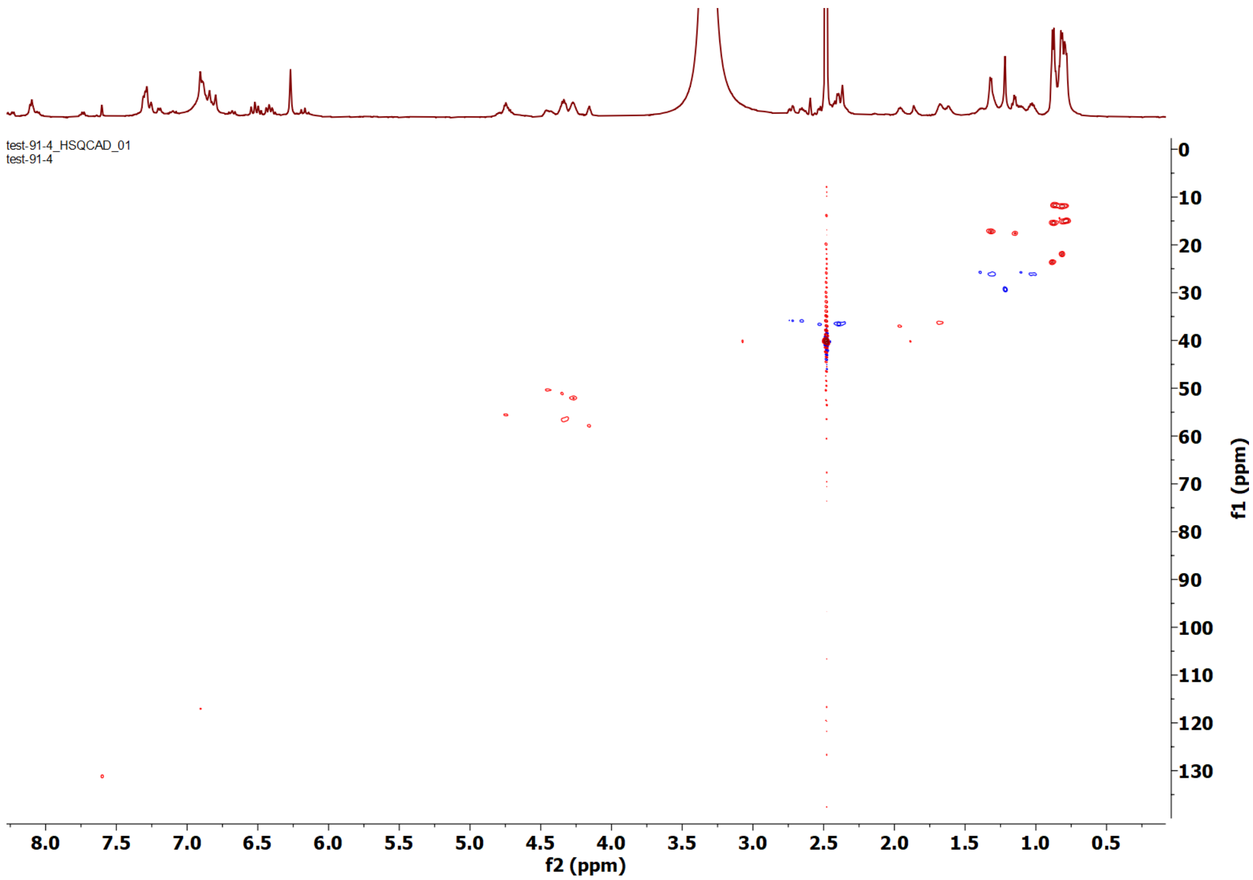

Figure S23. HSQC spectrum of Bromoalterochromide E/E' (**7**/**8**, 600 MHz, DMSO-d_6).

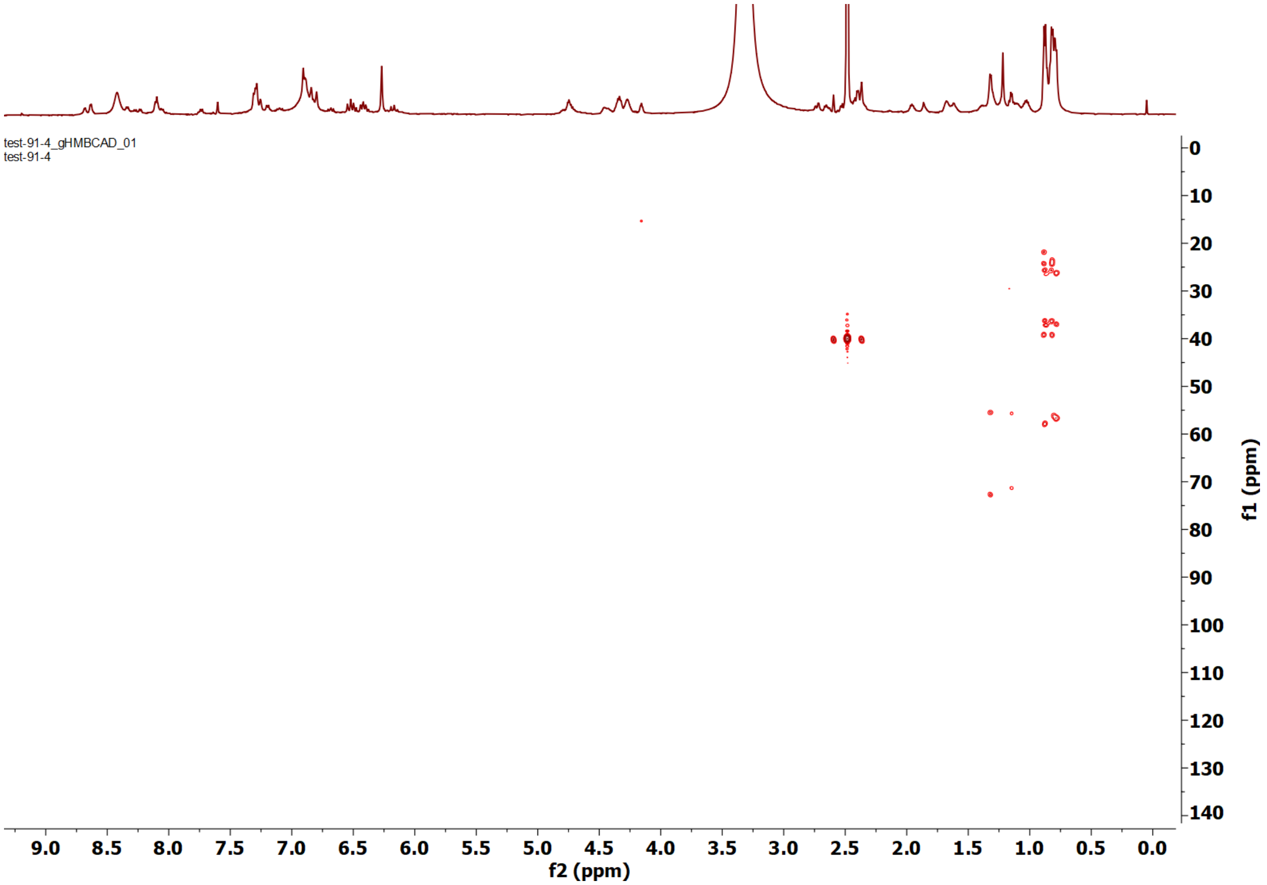

Figure S24. HMBC spectrum of bromoalterochromide E/E' (**7**/**8**, 600 MHz, DMSO-d_6).

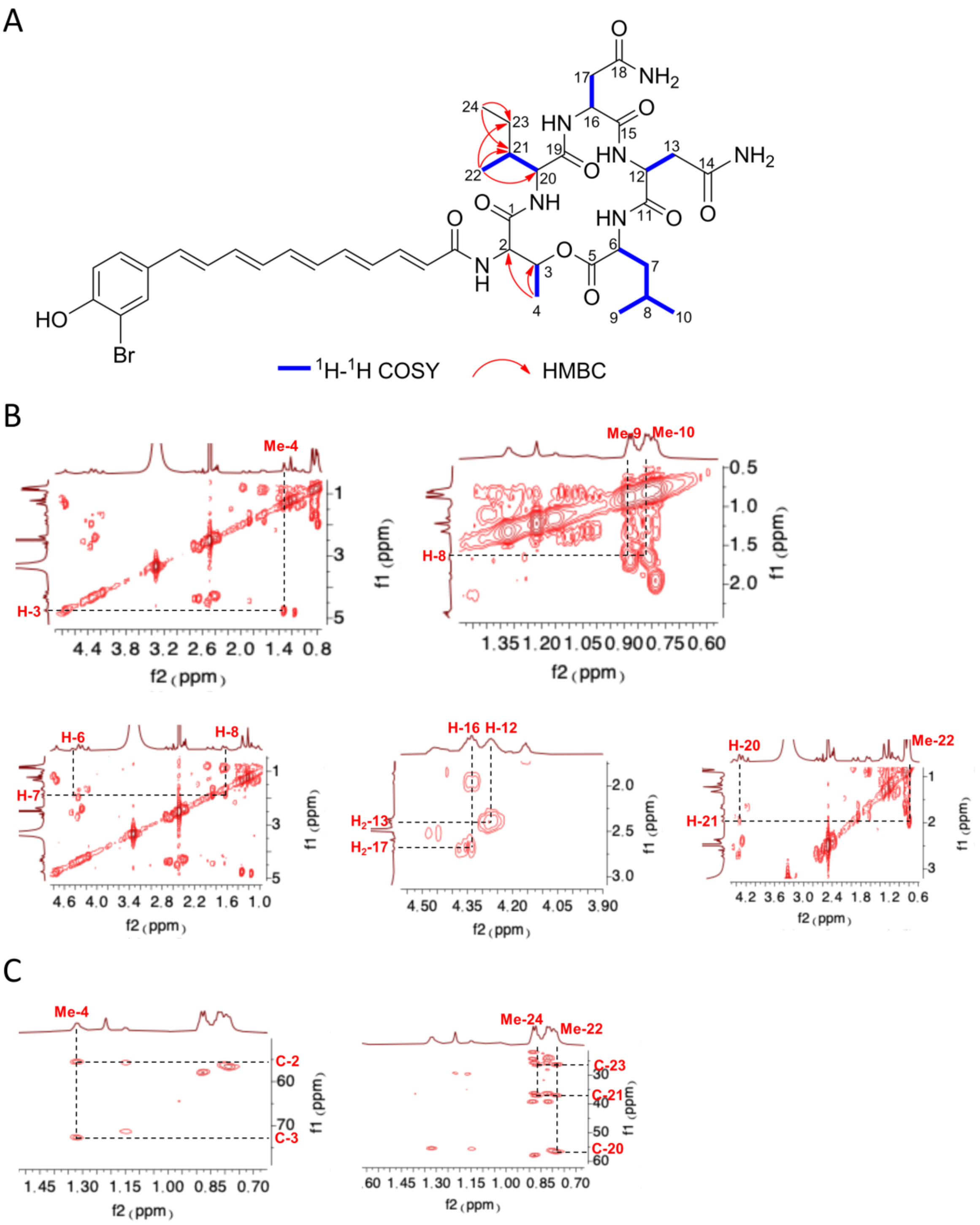

Figure S25. (**A**) Key ^{1}H-^{1}H COSY and HMBC correlations for bromoalterochromides E/E' (**7**/**8**). (**B**) Cross-peaks observed in ^{1}H-^{1}H COSY. (**C**) Cross-peaks observed in HMBC.

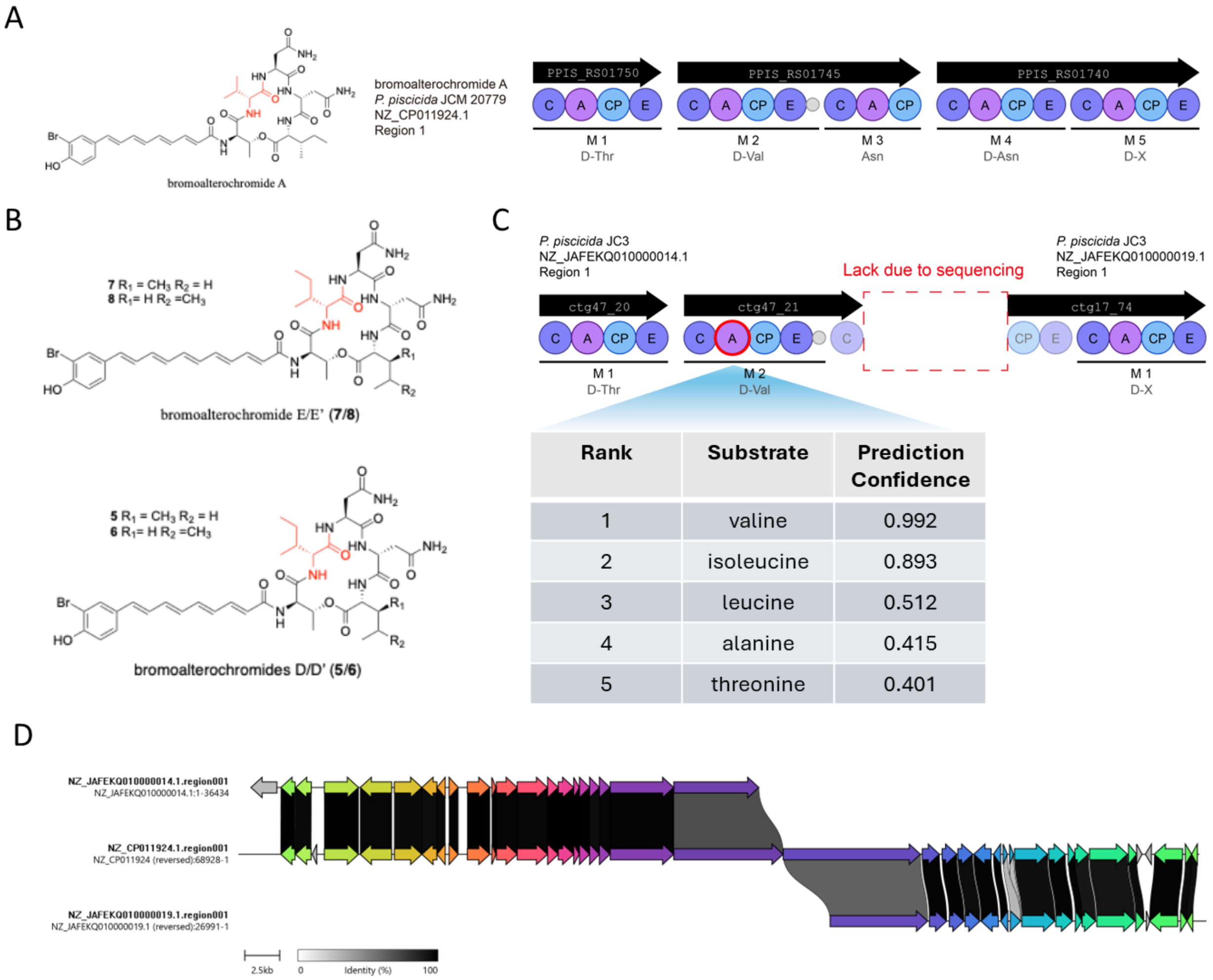

Rank	Substrate	Prediction Confidence
1	valine	0.992
2	isoleucine	0.893
3	leucine	0.512
4	alanine	0.415
5	threonine	0.401

Figure S26. Bioinformatics-guided stereochemical assignment of amino acids in bromoalterochromides E/E'. (**A**) Chemical structure of bromoalterochromide A and the nonribosomal peptide synthetase (NRPS) biosynthetic gene cluster (BGC) reported in *P. piscicida* JCM 20779. Five NRPS modules install the amino-acid residues of bromoalterochromide A. (**B**) Chemical structures of bromoalterochromides identified in this study from *P. piscicida* JC3. (**C**) Putative bromoalterochromide BGC in *P. piscicida* JC3 reconstructed from the draft genome. Two modules are absent due to incomplete sequencing. MS/MS, NMR, and Marfey's analyses support incorporation of one L-Asn and one D-Asn in the bromoalterochromides E/E' (**7**/**8**) and D/D' (**5**/**6**). Re-evaluation of adenylation (A)-domain substrate specificity with PARAS using the JC3 NRPS sequences indicates potential A-domain promiscuity in amino-acid recognition. This amino acid recognition aligns well with the structure elucidation from MS/MS and NMR. The E-domains are consistent with the established stereochemistry of the amino acid residues. A, adenylation domain; T, peptidyl carrier protein (thiolation) domain; C, condensation domain; E, epimerization domain. (D) Comparison of the candidate bromoalterochromide BGC. The top and bottom tracks are *P. piscicida* JC3 draft-genome contigs, and the middle track is *P. piscicida* JCM 20779 cluster. Conserved synteny across the NRPS core is maintained, whereas the JC3 contigs break within the module region; the two "missing" modules thus reflect assembly gaps rather than biological absence.

References

(1) Atencio, L. A.; Boya P., C. A.; Martin H., C.; Mejía, L. C.; Dorrestein, P. C.; Gutiérrez, M. Genome Mining, Microbial Interactions, and Molecular Networking Reveals New Dibromoalterochromides from Strains of *Pseudoalteromonas* of Coiba National Park-Panama. *Marine Drugs* **2020**, *18* (9), 456. https://doi.org/10.3390/md18090456.

(2) Ross, A. C.; Gulland, L. E. S.; Dorrestein, P. C.; Moore, B. S. Targeted Capture and Heterologous Expression of the *Pseudoalteromonas* Alterochromide Gene Cluster in *Escherichia coli* Represents a Promising Natural Product Exploratory Platform. *ACS Synth. Biol.* **2015**, *4* (4), 414–420. https://doi.org/10.1021/sb500280q.

(3) Suria, A. M.; Tan, K. C.; Kerwin, A. H.; Gitzel, L.; Abini-Agbomson, L.; Bertenshaw, J. M.; Sewell, J.; Nyholm, S. V.; Balunas, M. J. Hawaiian Bobtail Squid Symbionts Inhibit Marine Bacteria via Production of Specialized Metabolites, Including New Bromoalterochromides BAC-D/D′. *mSphere* **2020**, *5* (4), e00166-20. https://doi.org/10.1128/mSphere.00166-20s
